# Experiences and Outcomes of Using e-Prescribing for Opioids: Rapid Scoping Review

**DOI:** 10.2196/49173

**Published:** 2023-12-28

**Authors:** Sara J T Guilcher, Stephanie R Cimino, Mina Tadrous, Lisa M McCarthy, Jessica Riad, Andrea C Tricco, Simon Hagens, Jennifer Lien, Sukirtha Tharmalingam, Tara Gomes

**Affiliations:** 1 Leslie Dan Faculty of Pharmacy University of Toronto Toronto, ON Canada; 2 Institute for Better Health Trillium Health Partners Mississauga, ON Canada; 3 Institute of Health Policy, Management and Evaluation Dalla Lana School of Public Health University of Toronto Toronto, ON Canada; 4 Rehabilitation Sciences Institute Temerty Faculty of Medicine University of Toronto Toronto, ON Canada; 5 Epidemiology Division Dalla Lana School of Public Health University of Toronto Toronto, ON Canada; 6 Li Ka Shing Knowledge Institute St. Michael’s Hospital Unity Health Toronto, ON Canada; 7 Canada Health Infoway Toronto, ON Canada

**Keywords:** e-prescribing, opioid prescription, opioid use, rapid scoping review

## Abstract

**Background:**

e-Prescribing is designed to assist in facilitating safe and appropriate prescriptions for patients. Currently, it is unknown to what extent e-prescribing for opioids influences experiences and outcomes. To address this gap, a rapid scoping review was conducted.

**Objective:**

This rapid scoping review aims to (1) explore how e-prescribing has been used clinically; (2) examine the effects of e-prescribing on clinical outcomes, the patient or clinician experience, service delivery, and policy; and (3) identify current gaps in the present literature to inform future studies and recommendations.

**Methods:**

A rapid scoping review was conducted following the guidance of the JBI 2020 scoping review methodology and the World Health Organization guide to rapid reviews. A comprehensive literature search was completed by an expert librarian from inception until November 16, 2022. Three databases were electronically searched: MEDLINE (Ovid), Embase (Ovid), and Scopus (Elsevier). The search criteria were as follows: (1) e-prescribing programs targeted to the use or misuse of opioids, including those that were complemented or accompanied by clinically focused initiatives, and (2) a primary research study of experimental, quasi-experimental, observational, qualitative, or mixed methods design. An additional criterion of an ambulatory component of e-prescribing (eg, e-prescribing occurred upon discharge from acute care) was added at the full-text stage. No language limitations or filters were applied. All articles were double screened by trained reviewers. Gray literature was manually searched by a single reviewer. Data were synthesized using a descriptive approach.

**Results:**

Upon completing screening, 34 articles met the inclusion criteria: 32 (94%) peer-reviewed studies and 2 (6%) gray literature documents (1 thesis study and 1 report). All 33 studies had a quantitative component, with most highlighting e-prescribing from acute care settings to community settings (n=12, 36%). Only 1 (3%) of the 34 articles provided evidence on e-prescribing in a primary care setting. Minimal prescriber, pharmacist, and clinical population characteristics were reported. The main outcomes identified were related to opioid prescribing rates, alerts (eg, adverse drug events and drug-drug interactions), the quantity and duration of opioid prescriptions, the adoption of e-prescribing technology, attitudes toward e-prescribing, and potential challenges with the implementation of e-prescribing into clinical practice. e-Prescribing, including key features such as alerts and dose order sets, may reduce prescribing errors.

**Conclusions:**

This rapid scoping review highlights initial promising results with e-prescribing and opioid therapy management. It is important that future work explores the experience of prescribers, pharmacists, and patients using e-prescribing for opioid therapy management with an emphasis on prescribers in the community and primary care. Developing a common set of quality indicators for e-prescribing of opioids will help build a stronger evidence base. Understanding implementation considerations will be of importance as the technology is integrated into clinical practice and health systems.

## Introduction

### Background

Over the past decade, the rates of opioid-related harms have been increasing in North America [1-3]. Opioid-related harms may include opioid use disorder, adverse drug reactions and events, opioid toxicities, and death [4-7]. Since the early 2010s, there has been growing awareness of these potential opioid-related harms across North America [8,9]. Although most recent opioid-related harms are due to the unregulated drug supply (ie, fentanyl) [10-12], a long history of opioid prescribing practices for acute and chronic pain has contributed to harms [8]. In response to the increasing numbers of opioid-related harms in the United States and Canada, professional standards for opioid prescriptions were revised in 2016 and 2017, respectively, to include recommendations for more conservative opioid prescription practices [13-15].

e-Prescribing is designed to help facilitate the safe and appropriate prescribing of medications. e-Prescribing (in the Canadian context) is the secure electronic creation and transmission of a prescription between an authorized prescriber and a patient’s pharmacy of choice [16]. It uses clinical point-of-service solutions to integrate clinical workflow and software. e-Prescribing has shown some promising benefits at the patient, clinician, and health system levels. At the patient level, e-prescribing has improved patient safety [17-22] and patient experiences with accessing medications [23-25]; for example, the implementation of e-prescribing resulted in decreased rates of adverse drug events and prescribing errors [17-22]. It has also been shown to improve patient experience through easier access to medications and reduced wait times for dispensing [19,23,24]. At the clinician and health system levels, e-prescribing can improve workflow efficiency (eg, facilitating communication between prescribers and dispensers [26] and improve the rates of medication adherence, measured by prescriptions being filled [27-31]), resulting in both reduced health care costs and improved health outcomes [32,33]. Although these benefits of e-prescribing have been well described, there remains a gap in understanding the extent to which e-prescribing can influence safe and appropriate opioid use as well as clinically relevant experiences and outcomes.

### Objectives

To address this gap, a rapid scoping review was undertaken to answer the following question: *what are the direct impacts of e-prescribing for opioids on experiences and outcomes?* The specific objectives of this review were to (1) explore how e-prescribing has been used clinically with opioids; (2) examine the effects of e-prescribing of opioids on clinical outcomes, patient or clinician experience, service delivery, and policy; and (3) identify any gaps in the literature to inform future studies and recommendations.

## Methods

### Protocol and Registration

A rapid scoping review was conducted following the guidance of the JBI 2020 scoping review methodology [34] and the World Health Organization guide for rapid reviews [35]. Streamlined methods to conduct the rapid review followed the steps outlined by the Cochrane Rapid Reviews Methods Group in 2020 [36]. Reporting aligns with the PRISMA-ScR (Preferred Reporting Items for Systematic Reviews and Meta-Analyses extension for Scoping Reviews) statement [37]. The PRISMA-ScR checklist can be found in Multimedia Appendix 1 [37]. The protocol for this review was registered with OSF Registries [38].

### Eligibility Criteria

Eligibility criteria for the review evolved during the screening stages. During the title and abstract screening, inclusion criteria were as follows: (1) e-prescribing programs that were targeted to opioid use or misuse, including those that were accompanied or complemented by clinically focused initiatives, and (2) a primary research study of experimental (eg, randomized controlled trials), quasi-experimental (eg, nonrandomized controlled trials, controlled before-and-after studies, or interrupted time series), observational (eg, cohort studies, case-control studies, or cross-sectional studies), qualitative, or mixed method design. At the full-text screening phase, an additional inclusion criterion was added: an ambulatory component of e-prescribing (eg, e-prescribing of opioids occurred at discharge from acute care, in the emergency department, or in the community). This criterion was not included until the full-text stage to ensure that all relevant articles were included for review because abstracts were not likely to clearly specify the involvement of an ambulatory component. The exclusion criteria for all stages of peer-reviewed article screening included (1) prescribing that occurred within 1 hospital system (eg, within an acute care ward), (2) articles that did not look at the impact of e-prescribing on opioid use, (3) digital solutions for prescribing that did not include e-prescribing (eg, digital fax), (4) not a primary research study (eg, commentaries and opinion pieces), and (5) conference materials (eg, abstracts). Gray literature was included if the aforementioned criteria were met; however, articles were not required to be a research study.

### Information Sources

A literature search was conducted by an expert librarian (Leah M Boulos) on articles published from database inception until November 16, 2022. Three databases were electronically searched: MEDLINE (Ovid), Embase (Ovid), and Scopus (Elsevier). Gray literature was searched using a string of key terms in Google and ProQuest Dissertations & Theses Global. No filters or language limitations were applied.

### Search

The search strategies were developed based on 2 key concepts (e-prescribing and opioids) in consultation with the expert librarian who ran the search (Leah M Boulos). Previously published systematic reviews on opioids were also searched to identify relevant opioid-related terms [39-41]. Search strategies for the databases and gray literature can be found in Multimedia Appendix 2. A second librarian reviewed the search strategy using the Peer Review of Electronic Search Strategies (PRESS) checklist [42].

### Selection of Evidence Sources

Before deduplication, records from MEDLINE and Embase were removed from the Scopus database search using the AND NOT function to ensure that all relevant articles could be exported to EndNote (Clarivate); Scopus has a 2000-record export limit. Deduplication of the resulting list of articles from the 3 databases was then conducted in EndNote using the method developed by Bramer et al [43]. The literature review software, Covidence (Veritas Health Innovation Ltd), was used to streamline the article screening process. At the title and abstract screening phase, a pilot test using 20 articles was conducted by the reviewers (SRC, SJTG, JR, Shreya Mahajan, Shanzeh Chaudhry, and Alyssa Yang). After the pilot test, the team met to review the inclusion criteria, which were updated to ensure clarity. During this phase, eligibility criteria were kept broad to ensure that as many relevant articles were included as possible (eg, if there was uncertainty about the ambulatory component, articles were moved to full-text review). All articles were screened independently by 2 reviewers (SRC and JR), with any conflicts resolved through team discussion.

Once the title and abstract screening was completed, 10 full-text articles were randomly selected for pilot testing to ensure consistent application of the eligibility criteria across all reviewers. At this phase, articles that did not include an ambulatory component (ie, did not involve opioids prescribed at acute care discharge, in the emergency department, or in the community) were excluded. All full-text articles were independently screened by 2 of the 5 reviewers (SRC, JR, Shreya Mahajan, Shanzeh Chaudhry, Alyssa Yang) using the updated criteria, which included the ambulatory component.

Using Google, gray literature was manually searched by 1 reviewer (SRC). After reviewing the last relevant citation, an additional 20 citations were reviewed to ensure that all relevant materials were included. For dissertations and theses, this same process was completed by 2 reviewers (SRC and JR) using ProQuest Dissertations & Theses Global.

### Data Extraction and Charting Process

Data extraction, using the Covidence Data Extraction 2.0 form, was conducted once the full-text screening was completed. Key data that were collected from the articles included study characteristics, population characteristics adapted from the Cochrane PROGRESS-Plus equity variable recommendations [44] (sample size, age, sex, gender, ethnicity or race, religion, income, education, geographic location, and social capital), the description of e-prescribing (design, prescriber context, intended recipients, indication for opioids, and accompanied or not accompanied by clinically focused initiatives), study outcomes, and findings (eg, the descriptions of data-driven activities or analysis for managing the prescribing of opioids or informing better policy and interventions, opioid dependency, opioid-related death, health care use owing to opioids, economic costs owing to opioids, fraud, and the transparency of prescription history). A pilot test was conducted by 4 reviewers (JR, Shreya Mahajan, Shanzeh Chaudhry, and Alyssa Yang) with the extraction of 1 assigned article per person. Each of the pilot articles was spot-checked by an independent trained reviewer (SRC) to ensure consistency in extraction across reviewers. The remaining data extraction was conducted by the 4 reviewers (JR, Shreya Mahajan, Shanzeh Chaudhry, and Alyssa Yang), with quality checks conducted by the independent reviewer (SRC). A quality assessment of the articles was not conducted as per scoping review standards [34].

### Data Synthesis

The findings from the included articles were synthesized using descriptive approaches. Descriptive summaries of the study characteristics, population characteristics, study outcomes, and findings were conducted. Summaries of the findings were developed by collating study findings that reported on similar topics (eg, e-prescribing setting and the rates of prescribing opioids). Once the information was organized, a section header was developed based on the subject matter of each section. This organization process was carried out by 2 members of the authorship team (SRC and JR) in conjunction with members of the senior research team (SJTG, MT, LMM, and TG).

## Results

### Study Selection

**Figure 1:**
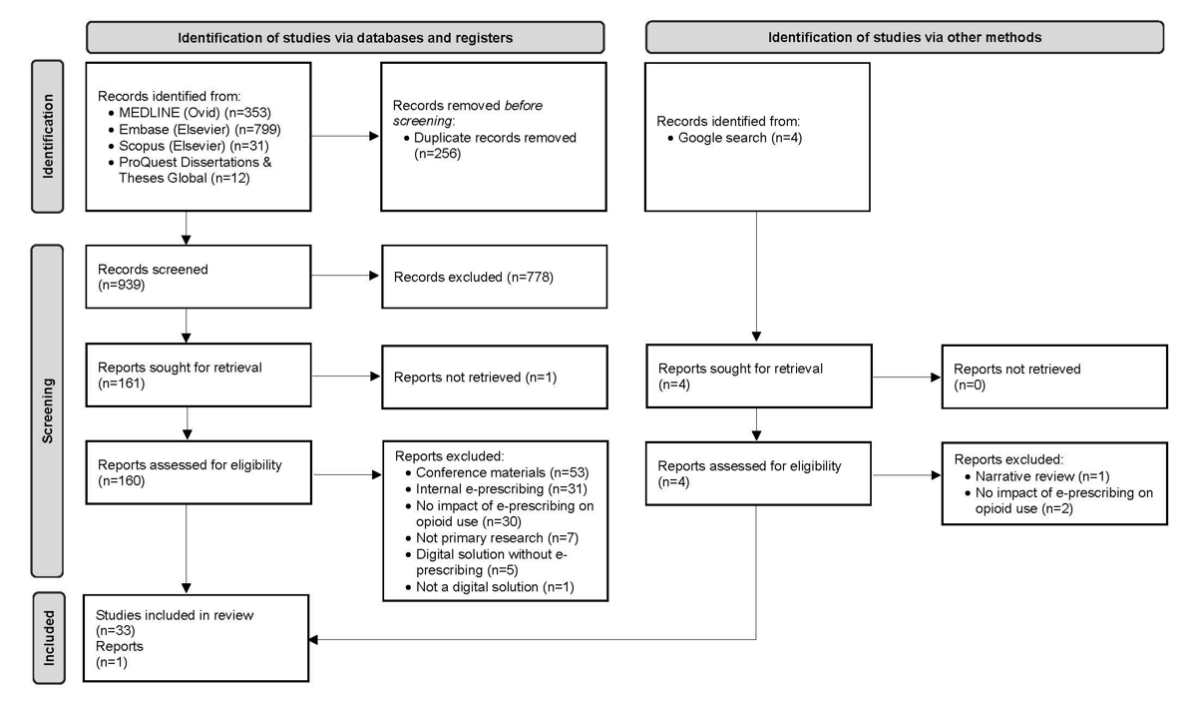
PRISMA (Preferred Reporting Items for Systematic Reviews and Meta-Analyses) 2020 flow diagram for new systematic reviews, which includes searches of databases, registers, and other sources.

The literature searches yielded 1183 articles (refer to Figure 1 for the PRISMA [Preferred Reporting Items for Systematic Reviews and Meta-Analyses] diagram). After the removal of duplicates from the 1183 articles, 939 (79.4%) were included in the title and abstract review. After this initial screening phase, 161 (17.1%) of the 939 reports were sought for retrieval; however, of the 161 articles, the full text of 1 (0.6%) article could not be retrieved, leaving 160 (99.4%) full-text articles assessed for eligibility. With respect to gray literature, 16 articles were identified: 12 (75%) dissertations or theses and 4 (25%) potentially relevant reports found via Google. After the screening of the total 176 articles, 32 (18.2%) full-text articles [28,45-75] and 2 (1.1%) gray literature documents (thesis: n=1, 50%; report: n=1, 50%) [76,77] met the inclusion criteria and were included in the rapid review. The characteristics of the identified studies (32 full-text studies and 1 thesis) are described in the following subsections, followed by a description of the gray literature report.

### Study Characteristics

#### Geographic Distribution of Studies

The 33 identified studies were mostly conducted in the United States (n=25, 76%) [28,46-49,51-54,56-58,61,63-65,67-71, 73-75,77], followed by Canada (n=2, 6%) [59,60], Australia (n=1, 3%) [66], and Brazil (n=1, 3%) [62] (Table 1). Publication dates ranged from 2005 to 2022.

**Table 1:** Characteristics of included articles (n=33)^a^.

Authors, year; country	Study design	Main outcome related to opioid use and e-prescribing	e-Prescribing setting	Key findings and results related to opioid use and e-prescribing
Abdel-Qader et al [45], 2010; United Kingdom	Cross-sectional	Prescribing errors	Discharge (acute care)	The most frequently recorded individual medications associated with an error included codeine (n=18, 2.9%). The most frequent high-risk medications (associated with erroneous orders) included codeine (n=18, 22.2%) and morphine (n=7, 8.6%).
Ancker et al [46], 2021; United States	Quality improvement	Proportion of guideline- concordant (contained ≤12 pills, ie, a 3-d supply) prescriptions and number of mouse clicks and keystrokes to place order	Ambulatory^b^	At Weill Cornell Medicine, guideline-concordant prescriptions immediately rose from an average of 12% to 31% of all prescriptions. At the Institute for Family Health, guideline-concordant prescriptions remained at 44%. The intervention (to test the effect of a default prescription order intervention on opioid prescribing choices) was not associated with any change in the total volume of opioid prescriptions. There was a 62.7% decrease in total keystrokes (3552 in the 6-mo period before the default prescription order intervention to 1323 in the 6-mo period afterward).
Ariosto [77], 2011; United States	Retrospective	Prescribing rate for prescriptions with allergy alerts triggered and overwritten	Discharge (acute care)	The override rate for the patients’ first opiate alerts was 89%. Opiate allergy override rate was 93% for all admissions and readmissions. More than half of all discharges had opiates ordered during their stay, and of these, among patients with recorded opiate allergies (9.1%), 25,461 CPOE^c^ opiate allergy alerts were triggered. Override rates remained high, with 80% for advanced practice nurses and 90% for physicians, with advanced practice nurses less likely to override the patient’s first opiate alert than physicians (*P*=.001).
Bicket et al [47], 2017; United States	Retrospective	Prescribing rate and errors	Ambulatory	The most prescribed opioid was oxycodone IR^d^ (71%) with other opioids being prescribed less often (hydromorphone IR: 10%, morphine IR: 3%, oxycodone CR^e^: 3%, fentanyl patches: 3%, tramadol IR: 3%, and morphine CR: 2%). Tablet form was the most common formulation of opioid prescriptions for adults (92%). A similar number of handwritten (47%) and hospital computer–generated (47%) prescriptions was found for the opioid prescriptions; however, fewer prescriptions were generated by non–hospital computer software (7%). All prescriptions containing a best practice deviation or lacking 2 patient identifiers were handwritten and not computer generated.
Chiu et al [48], 2018; United States	Pre-post intervention study	Prescribing quantity and dose and refill rate	Outpatient department (surgical)	The median number of opioid pills prescribed decreased from 30 to 20/prescription after implementation (*P*<.001). The percentage of prescriptions written for 30 pills decreased, from before to after the default change, from 39.7% to 12.9% (*P*<.001). The percentage of prescriptions written for 12 pills increased, from before to after the default change, from 2.1% to 24.6% (*P*<.001). No statistical difference was found in opioid refill rates from before to after the default change (3% vs 1.5%; *P*=.41). Results from adjusted linear regression analysis indicated that the number of opioid pills decreased by 5.22 (95% CI −6.12 to −4.32) per prescription. After the default change, total opioid MME^f^ prescribed decreased by 34.41 (95% CI −41.36 to −27.47) after the default change.
Danovich et al [49], 2019; United States	Descriptive	Prescribing rate	Emergency department	Between the pre- and postimplementation stages of the New York state e-prescribing mandate, there was an absolute decrease of 724 (53%) opioid prescriptions (1366 vs 642; *P*<.001), which is an absolute difference of 2.3% (95% CI 2%-2.6%).
Delgado Sánchez et al [50], 2005 ; Spain	Prospective	Prescribing errors	Hospital pharmacy	Of 1183 prescription and transcription errors, 62 (5.24%) involved opioid pain relievers.
Everson et al [51], 2020; United States	Retrospective	Prescribing rates	Not reported	The population-weighted percentage of opioids prescribed using EPCS^g^ increased from 0% in 2013 to 27% in 2018. From 2013 to 2018, the national rates of opioid prescriptions decreased from 78 to 53 prescriptions/100 persons. By 2018, EPCS increased to 69.4% in mandated states and 23.6% in nonmandated states. In multivariable models, it was found that a 10 percentage-point increase in the use of EPCS was associated with an additional 2 prescriptions/100 persons (95% CI 1.3-2.8) and a 0.8% (95% CI 0.06%-1.5%) increase in MME/100 persons.
Fischer et al [28], 2011; United States	Retrospective	Primary nonadherence	Outpatient department	Of all e-prescriptions, opioids made up 3%. The rate of primary nonadherence for opioids was 23.9%.
George et al [52], 2016; United States	Descriptive	Prescribing trends and errors	Discharge (pediatric)	The most prescribed opioid was oxycodone (uncombined; 73%). Codeine was prescribed in combination with acetaminophen (7%). Liquid formulations were prescribed to 98% of children aged <6 y and 16% of children aged >12 y. A subset of 700 regenerated prescriptions were legible (drug, amount dispensed, dose, patient demographics, and provider name) and used best prescribing practice. Of the 700 regenerated prescriptions, 25 had incorrect weights; 14 varied by ≤10%, 2 varied by >15%, 1 resulted in underdosing, and 1 in overdosing.
Griffey et al [53], 2012; United States	Prospective controlled trial	Medication ordering consistent with recommendations	Emergency department	There was a significant difference in agreement with recommendations between the on and off periods (36% vs 26%; *P*<.001) for opioids. Hydromorphone was the second most common drug that was written at 10-fold dosing orders (10 times the preferred dose; 6 of 38 orders).
Hickman et al [54], 2018; United States	Retrospective	Prescribing errors	Outpatient department	Top reasons for the discontinued erroneous orders were medication ordered for the wrong patient (n=60, 27.8%), wrong drug ordered (n=40, 18.5%), and duplicate order placed (n=31, 14.4%). Oxycodone was the most frequent drug discontinued error (3%).
Hung et al [55], 2021; Taiwan	Pre-post intervention study	Prescribing errors	Discharge	Morphine was the third most common potential duplicated medication for the nervous system category (n=2472, 3.8%) after the intervention.
Jones et al [56], 2021; United States	Quality improvement	Provider compliance and prescribing quantity	Discharge (pediatric surgery)	Compliance of >90% with the opioid guidelines was achieved and sustained for 20 mo. There was a 54% reduction in opioids prescribed, from 71 MME/patient to 33 MME/patient in opioids prescribed, and the reduction was sustained for 12 mo.
Kearney et al [57], 2022; United States	Quality improvement	Prescribing compliance to pill quantities and MME	Discharge (surgical)	The mean compliance with prescribing at or below the suggested opioid pill quantities and MMEs improved by <5%. After the implementation of the prescribing tool, the number of MMEs prescribed significantly decreased by 26% (100 vs 75 MME) in a subgroup of hand surgeries (*P*<.001).
Kim et al [58], 2017; United States	Before-after comparison study	Recommended dose rate	Emergency department	The recommended dosing of opioids significantly increased after the implementation of default geriatric dosing in the CPOE template (29% vs 35.2%; *P*<.001). Of the opioids, fentanyl (adjusted risk difference 13%, 95% CI 2%-23%), morphine (adjusted risk difference 11%, 95% CI 4%-19%), and hydromorphone (adjusted risk difference 7%, 95% CI 4%-10%) showed the greatest increases.
Kurteva et al [59], 2021; Canada	Prospective	Prescribing errors	Discharge (acute care)	A total of 1530 (43.89%) of 3486 patients were prescribed opioids, of which 205 (13.4%) patients had at least 1 opioid-related medication error. There was a 69% lower risk of having an opioid medication error when the discharge prescription was finalized with the electronic reconciliation software (adjusted odds ratio 0.31, 95% CI 0.14-0.65). The medication error rate is higher for handwritten prescriptions than for e-prescriptions (20.6% vs 1.2%). There is a 2.3 times increased risk of health care use in the 30-d postdischarge period associated with opioid-related medication errors (adjusted odds ratio 2.32, 95% CI 1.24-4.32).
Leung et al [60], 2013; Canada	Quasi-experimental	Rate of preventable ADEs^h^	Discharge (renal failure)	Preventable ADEs for opioids decreased significantly from before to after the implementation of CPOE systems with clinical decision support (28 vs 4; *P*=.002) but not nonpreventable ADEs (1 vs 5; *P*=.15).
McPhillips et al [61], 2005; United States	Retrospective	Potential drug errors	Ambulatory (pediatrics)	Of the analgesic dispensing events, 15% were above the maximum recommended dose, with most occurring for oxycodone (28 of 51 potential overdoses). Of the 18 dispensing events associated with potential overdosing in adolescents, 17 were for oxycodone.
Moura et al [62], 2012; Brazil	Quasi-experimental	DDI^i^ rates	Hospital pharmacy	Incident rate/1000 patient-d for high-severity DDI pair amiodarone-fentanyl was not significantly different before and after the intervention (0.36 vs 0.18; *P*=.99). Overall, there was a 71% reduction in high-severity DDIs (*P*<.01).
Ney and Weathers [63], 2019; United States	Cross-sectional	Prescribing rate	Ambulatory (primary care)	Comparing physicians with access to CPOE and those without, opiates were prescribed 10.4% of the time compared with 7.5%. The adjusted odds of opiate prescription were significantly greater in visits to physicians who had access to CPOE (odds ratio 1.35, 95% CI 1.14-1.58; *P*=.001). Among patients citing pain, the adjusted odds of opioid prescription were significantly greater when physicians had access to CPOE compared with those without (odds ratio 1.28, 95% CI 1.02-1.61; *P*=.04).
Ramaseshan et al [64], 2020; United States	Prospective	PDNU^j^, refill rate, and pain scores	Discharge (surgical)	The median PDNU was 24.0 (IQR 0-82.5) MME (equivalent to <4 oxycodone 5 mg tablets or 5 hydrocodone 5 mg tablets). Approximately 75% of the patients required <11 oxycodone tablets. Nearly one-third of the patients (29.2%) did not use any narcotics after discharge. Median unused MME was 90.0 (IQR 45-112.5). At the postoperative week 1 and postoperative weeks 4-6 time points, approximately 88.5% of the patients felt that their prescribed narcotic amount was sufficient for their pain needs. A minority of the patients (10.6%) needed a narcotic refill.
Santistevan et al [65], 2018; United States	Retrospective	Prescribing rate and quantity	Emergency department	Before the intervention, 4104 adult patients received opioid discharge prescriptions, and 2464 received them after the intervention. The median quantity of opioid tablets prescribed decreased from 20 to 15 (*P*<.001) after the removal of the default quantity. The proportion of patients receiving 20 tablets was reduced from 0.5 (95% CI 0.48-0.52) to 0.23 (95% CI 0.21-0.24) after default quantity removal (*P*<.001), despite 20 tablets being the most frequent quantity of tablets received in both groups.
Schwartz et al [66], 2019; Australia	Descriptive	Prescribing quantity	Emergency department	Oxycodone quantity of 5 tablets increased from 3% to 32% after the intervention. Oxycodone quantity of 20 tablets fell from 40% to 24% after the intervention. The mean number of oxycodone tablets prescribed/patient fell from 13.8 (SD 5.1) to 10.8 (SD 5.6). Paracetamol with codeine quantity of 10 tablets increased from 2% to 24%, whereas it fell from 98% to 76% for quantity of 20 tablets. The mean number of paracetamol with codeine tablets prescribed/patient fell from 19.8 (SD 1.5) to 17.6 (SD 4.2).
Shoji et al [67], 2022; United States	Retrospective	Prescribing rate and amount	Outpatient department	Significant decrease in MME prescribed for ganglion excision (*P*=.03) and carpometacarpal arthroplasty (*P*<.01). Significant decrease in the total number of tablets prescribed for ganglion excision (*P*<.01), carpometacarpal arthroplasty (*P*<.01), and distal radius fracture open reduction internal fixation (*P*=.04). No significant decrease in amount of opioid tablets (*P*=.27) or average MME (*P*=.44) for carpal tunnel release. Across the whole population, there was a significant increase in the number of patients not receiving opioid prescriptions after surgery (*P*<.01).
Siff et al [68], 2021; United States	Prospective	Prescribing rates	Outpatient department	General medicine (adult, pediatric, and family) accounted for 41% of the opioid prescriptions and surgery accounted for 23%. Opioid prescriptions with overridden naloxone prompts were due to the following reasons: 57% naloxone not indicated, 30% of the patients declined naloxone, 4% of the patients already had a prescription for naloxone, and 9% other.
Slovis et al [69], 2021; United States	Quasi-experimental	Prescribing quantity and duration	Discharge (outpatient department)	Overall median quantity of opioid tablets dispensed before vs after the intervention was significantly reduced (54 vs 42; *P*<.001). Median duration of opioid treatment significantly reduced (10.5 d vs 7.5 d; *P*<.001). There were small but significant reductions in the proportion of prescriptions for morphine (6.3% to 5.95%; *P*=.04) and oxymorphone (0.37% to 0.24%; *P*=.002). Although there was no change in the median 45 MMEs/d/prescription before and after the intervention, there was a significant reduction in the proportion of prescriptions for >90 MMEs/d (27.46% vs 22.86%; *P*<.001).
Thomas et al [71], 2012; United States	Cross-sectional	Expectations of EPCS	Ambulatory	Although many prescribers reported recurrent technical issues with their system, 76% felt comfortable with their e-prescribing system. The features most frequently used by prescribers were automated renewals (59.8% used it >1/d) and viewing prescribing (52.5% used it >1/d). Comparing users and nonusers of EPCS, users were more likely to expect EPCS to improve work flow and practice efficiency (69.6% vs 58.8%; *P*<.01), improve the management of pharmaceutical therapy within the practice (74.3% vs 58.1%; *P*<.01), and be easy to use (69.6% vs 54.8%; *P*=.02); users were also less likely to expect EPCS to cause system breaches of patient confidentiality (6.9% vs 14.7%; *P*=.05) or involve a learning curve that is disruptive to the practice (14.7% vs 33.4%; *P*<.001). Although certain security measures were seen as a burden and potential barrier, prescribers viewed EPCS as a tool to improve their practice.
Thomas et al [70], 2013; United States	Cross-sectional	Adoption, attitudes, and challenges after EPCS implementation	Community pharmacy	A majority (62%) of the total CSk prescriptions (electronic and paper) were electronically sent to prescribers. Prescribers found that EPCS was easy to use (72.9%), improved the accuracy of prescriptions (69.5%), improved workflow (66.1%), improved the monitoring of medications in the practice (59.3%), improved coordination with pharmacists (55.9%), and led to fewer calls to pharmacists (54.2%). However, the EPCS experience did not meet the high expectations reported before implementation. Providers using EPCS reported that safety problems (eg, prescribing errors) occurred less often after the EPCS implementation. Barriers included limited pharmacy participation and the unreliability of the technology.
Tora et al [72], 2014; Sweden	Prospective	Prevalence of drug-related problem	Discharge	Tramadol accounted for 1.6% of all alerts and had 1 of the highest proportions of alerts in comparison with other drugs (proportion frequency alert/frequency all drugs): 1.92).
Watterson et al [73], 2022; United States	Prospective	Successful discontinuation and time difference between discontinuation in clinic or pharmacy	Discharge (acute care)	After the implementation of CancelRx (discontinuation e-prescribing tool), there was an immediate and significant (*P*<.001) increase in the number of controlled substance medications that were successfully discontinued at the pharmacy after being discontinued in the clinic. A year after the implementation, the change was sustained (slope=0.03 percentage point, 95% CI −0.050 to 0.110) and did not revert to pre-CancelRx levels. After the CancelRx implementation, medication discontinuations in the pharmacy and clinic were all completed on the same day (all values=0) with a stable trend and almost no variation.
Weingart et al [75], 2014; United States	Prospective	Clinician behavior responding to alerts	Ambulatory	The majority (68.1%) of the antiemetic-triggered alerts were attributed to their interactions with analgesic opioids. Prescribers sometimes canceled the new order when an alert indicated an interaction between antiemetics and opioid analgesics, antiarrhythmics, and antidepressants. Prescribers were often prompted to cancel the order when there was an interaction between opioids and antiretrovirals, antiparkinson medications, antibiotics, antidepressants, and antineoplastic agents.
Weingart et al [74], 2009; United States	Retrospective	ADE alerts	Ambulatory	DDI alerts involving narcotic-narcotic and narcotic-benzodiazepine anticonvulsant combinations were judged to have prevented serious ADEs (2 for acetaminophen-propoxyphene combination with acetaminophen-hydrocodone combination annually and 1 for acetaminophen-propoxyphene combination with lorazepam annually).

^a^Only research studies are included in this table; the grey literature report is not reflected in this table owing to inability to extract the relevant information.

^b^Ambulatory was defined as e-prescribing occurring outside of a single system (eg, within a single hospital system). Prescriptions within the emergency department, outpatient department, and during transitions of care were included.

^c^CPOE: computerized physician order entry.

^d^IR: immediate release.

^e^CR: continuous release.

^f^MME: morphine milligram equivalent.

^g^EPCS: electronic prescribing for controlled substances.

^h^ADE: adverse drug event.

^i^DDI: drug-drug interaction.

^j^PDNU: postdischarge narcotic use.

#### Study Designs

All 33 studies had a quantitative component, with 3 (9%) being mixed methods studies [54,70,71]. The most common study designs were retrospective studies (9/33, 27%) [28,47,51,54,61,65,67,74,77] and prospective cohort studies (7/33, 21%) [50,59,64,68,72,73,75], followed by cross-sectional studies (4/33, 12%) [45,63,70,71], descriptive studies (3/33, 9%) [49,52,66], pre-post studies (3/33, 9%) [48,55,58], quasi-experimental studies (3/33, 9%) [60,62,69], quality improvement studies (3/33, 9%) [46,56,57], and a prospective controlled study (1/33, 3%) [53].

### Data Collection Methods

Data were obtained through a variety of collection methods, with the most common being electronic medical records (13/33, 39%) [45-49,54-58,60,66,77] and hospital or health care setting databases (12/33, 36%) [52,53,59,61,62,64,67-69,72-74]. Other data were obtained through a variety of methods and approaches, such as surveys (5/33, 15%) [54,63,66,70,71], structured interviews (2/33, 6%) [59,66], opioid prescribing rate maps (1/33, 3%) [51], iScribe (an e-prescribing system used for outpatient settings; 1/33, 3%) [28], data from the US Drug Enforcement Administration’s Automation of Reports and Consolidated Orders System (1/33, 3%) [51], a computer-generated data set (1/33, 3%) [65], a large pharmacy benefits management company (1/33, 3%) [28], and treatment orders (1/33, 3%) [50].

### Study Populations and Settings

With respect to the populations being studied (Table 2), most were clinical populations (24/33, 73%) [45-60,62,64,67,69,72,73,75,77], the general population (7/33, 21%) [28,61,63,65,66,68,74], and clinical prescribers (2/33, 6%) [70,71]. e-Prescribing settings varied among the included studies, with ambulatory settings (eg, emergency department and outpatient department) being the most common (16/33, 48%) [28,46-49,53,54,58,61,65-68,71,74,75]. Other settings included acute care discharge (12/33, 36%) [45,52,55-57,59,60,64,69,72,73,77], hospital pharmacy (2/33, 6%) [50,62], community pharmacy (1/33, 3%) [71], and primary care (1/33, 3%) [63]. Of the 33 studies, 1 (3%) did not report the setting [51].

Among the 13 studies that specified participant age, 7 (54%) studied adults (aged ≥18 y) [57,59,60,62,64-66], 3 (23%) studied a population comprising older adults (aged ≥65 y) [53,55,58], and 3 (23%) included a pediatric population (aged <18 y) [52,56,61]. Of the 33 articles, 16 (48%) [45,46,48,49,53,56,58-60,62-64,67,69,71,72] reported the sex of the participants (most were male). A few studies reported ethnicity or race (10/33, 30%) [46,48,49,53,60,63,64,67,71,77], comorbidities (5/33, 15%) [45,59,63,66,67], gender (3/33, 9%) [28,66,77], marital status (1/33, 3%) [64], employment status (1/33, 3%) [64], or geographic location (1/33, 3%) [63]. Income, education, the place of residence, social capital, and religion were not reported.

**Table 2:** Summary of participant demographics from the included articles (n=33)^a^.

Authors, year; country	Sample size	Sample demographics and clinical characteristics							
		Age	Sex	Gender	Ethnicity or race	Comorbidities	Marital status	Employment status	Geographic location
Abdel-Qader et al [45], 2010; United Kingdom	People: 1038	NR^b^	Female 52%; male 48%	NR	NR	Of 212 patients, 188 (88.7%) with prescribing errors	NR	NR	NR
Ancker et al [46], 2021; United States	Patients: 22,113 (Weill Cornell Medicine: n=18,218; The Institute for Family Health: n=3895)	NR	Weill Cornell Medicine: female 49.4% (n=9139); The Institute for Family Health: female 68.6% (n=2705)	NR	Weill Cornell Medicine: White 19.2% (n=3562); unknown race 59.9% (n=11,091); The Institute for Family Health: White 68.6% (n=1639)	NR	NR	NR	NR
Ariosto [77], 2011; United States	Patients: 30,321; alerts: 2767	Override: mean age 54.5 (SD 16.4) y; no override: mean age 54.7 (SD 16.7) y	NR	Female 69% (n=1900); male 31% (n=867)	Black 11% (n=302); White 86% (n=2385); other 3% (n=80)	NR	NR	NR	NR
Bicket et al [47], 2017; United States	Patients: 451	Mean age 47.5 (SD 17.4; range 18-100) y	NR	NR	NR	NR	NR	NR	NR
Chiu et al [48], 2018; United States	Patients: 2910	Before implementation: mean age 54.4 (SD 17.3) y; after implementation: mean age 54.5 (SD 16.4) y	Before implementation: male 33.1% (n=479); female 66.9% (n=968); after implementation: male 33% (n=483); female 67% (n=980)	NR	African American 10.9% (n=160); Asian 2.2% (n=32); Hispanic 15.3% (n=224); White 70.3% (n=1028); unknown 1.3% (n=19)	NR	NR	NR	NR
Danovich et al [49], 2019; United States	Patients: 44,626	Before implementation: mean age 47.5 (SD 16.7) y; after implementation: mean age 48.2 (SD 16.8) y	Before implementation: male 48%; female 52%; after implementation: male 54%; female 46%	NR	Before implementation: Asian 3.5%; Black 14.9%; White 62.8%; other 18.7%; after implementation: Asian 3.4%; Black 11.7%; White 68.7%; other 16.2%	NR	NR	NR	NR
Delgado Sánchez et al [50], 2005; Spain	Treatment orders: 41,931	NR	NR	NR	NR	NR	NR	NR	NR
Everson et al [51], 2020; United States	Observations: 459	NR	NR	NR	NR	NR	NR	NR	NR
Fischer et al [28], 2011; United States	Patients: 280,081; prescribers: 3634	Age-wise categories: <1 y (n=1108, 0.4%); 1-18 y (n=42,372, 15.1%); 19-44 y (n=68,449, 24.4%); 45-54 y (n=53,147, 19%); 55-65 y (n=60,611, 21.6%); >65 y (n=54,389, 19.4%)	NR	Male 39.6% (n=111,003); female 60.3% (n=169,021)	NR	NR	NR	NR	NR
George et al [52], 2016; United States	CS^c^ discharge pediatric prescriptions: 4218	Mean age 9 (SD 6.1; range 0-21) y	NR	NR	NR	NR	NR	NR	NR
Griffey et al [53], 2012; United States	Patients: 1407; orders: 2398	Intervention: mean age 74 (SD 7.4) y; control: mean age 75 (SD 7.2) y	Intervention: female 61%; control: female 60%	NR	Intervention: African American 15%; Hispanic 12%; White 69%; other 4%; control: African American 16%; Hispanic 10%; White 70%; other 4%	NR	NR	NR	NR
Hickman et al [54], 2018; United States	Prescriber responses: 312	NR	NR	NR	NR	NR	NR	NR	NR
Hung et al [55], 2021; Taiwan	Prescriptions: 1,719,478	NR	NR	NR	NR	NR	NR	NR	NR
Jones et al [56], 2021; United States	Surgeries: 5776	Median age 13 (IQR 9-16) y	Male 53%; female 47%	NR	NR	NR	NR	NR	NR
Kearney et al [57], 2022; United States	Surgeries: 1208; patients: 1134	NR	NR	NR	NR	NR	NR	NR	NR
Kim et al [58], 2017; United States	Patients: 1946	Before implementation: mean age 73.3 (SD 7.5) y; after implementation: mean age 73.1 (SD 7.4) y	Before implementation: female 49.6% (n=497); male 50.4% (n=505); after implementation: female 46% (n=434); male 54% (n=510)	NR	NR	NR	NR	NR	NR
Kurteva et al [59], 2021; Canada	Patients: 3486 (opioid on discharge: n=1530; no opioid on discharge: n=1956)	Opioid on discharge: mean age 66.6 (SD 13) y; no opioid on discharge: mean age: 71.8 (SD 15.5) y	Opioid on discharge: male 60.6% (n=927); no opioid on discharge: male 55.4% (n=1083)	NR	NR	Top 3 for opioid on discharge: cardiovascular disease 49.5% (n=968); pain syndromes 39.5% (n=604); cancer 30.4% (n=595)	NR	NR	NR
Leung et al [60], 2013; Canada	Patients: 815	Mean age 72.2 (range 18.0-102.0) y	Male 57% (n=427); female 43%: (n=321)	NR	African American 6% (n=45); Asian 1.7% (n=13); Hispanic 3.3% (n=25); Caucasian 87.4% (n=654); other 0.94% (n=7); not recorded 0.53% (n=4)	NR	NR	NR	NR
McPhillips et al [61], 2005; United States	Patients: 1933	NR	NR	NR	NR	NR	NR	NR	NR
Moura et al [62], 2012; Brazil	Patients: 2147	Phase 1: mean age: 52.7 (SD 20.9) y; phase 2: mean age 53.4 (SD 21.3) y	Phase 1: male 56% (n=1032); phase 2: male 36% (n=105)	NR	NR	NR	NR	NR	NR
Ney and Weathers [63], 2019; United States	Office-based medical visits: 233,390	CPOE^d^: age 0-17 y (17%); 18-64 y (53%); ≥65 y (30%); no CPOE: age 0-17 y (20%); 18-64 y (57%); ≥65 y (23%)	CPOE: female 58%; no CPOE: female 58%	NR	Asian or Native American 6%; Black 10%; Hispanic 13%; White 72%	Noncancer pain 23%; cancer 7%; chronic issue 40%	NR	NR	Physician: northeast (19%); midwest (20%); south (37%); west (23%); rural (2%)
Ramaseshan et al [64], 2020; United States	113 people	mean age 63.2 (SD 11.0) y	Female 100%	NR	African American 3.5% (n=4); Hispanic 7.1% (n=8); White 89.4% (n=101); non-Hispanic 92.9% (n=105); other 7.1% (n=8)	NR	Single 8% (n=9); married or partnership 70.8% (n=80); divorced 8% (n=9); widowed 10.6% (n=12)	Employed 46% (n=52); unemployed 8% (n=9); retired 37.2% (n=42); unknown 8% (n=9)	NR
Santistevan et al [65], 2018; United States	Adult patients: 6478	NR	NR	NR	NR	NR	NR	NR	NR
Schwartz et al [66], 2019; Australia	Patients: 208	Before implementation: mean age 49 (SD 17) y; after implementation: mean age: 44 (SD 15) y	NR	Before implementation: male 51% (n=52); after implementation: male 57% (n=60)	NR	Acute injury 31% (n=32); acute pain without injury 43% (n=44); renal colic 8% (n=8); chronic pain 17% (n=17); cancer-related pain 1% (n=1)	NR	NR	NR
Shoji et al [67], 2022; United States	Patients: 428	Before implementation: mean age 58 (SD 16) y; after implementation: mean age 57 (SD 15) y	Before implementation: female 72% (n=156); male 28% (n=60); after implementation: female 75% (n=159); male 25% (n=53)	NR	Asian 3.2% (n=7); Black 12% (n=25); Hispanic 9.3% (n=20); White 66% (n=142); other or NR 10% (n=22)	Chronic pain: no 92% (n=199); yes 8% (n=17)	NR	NR	NR
Siff et al [68], 2021; United States	Opioid prescriptions: 82,463	NR	NR	NR	NR	NR	NR	NR	NR
Slovis et al [69], 2021; United States	Patients: 30,975; prescriptions: 78,246	Median age 59 y	Female 56% (n=17,344)	NR	NR	NR	NR	NR	NR
Thomas et al [71], 2012; United States	Prescribers: 246	Mean age 52 y	Male 63%; female 37%	NR	White, Hispanic or Latino 2.5%; White, non-Hispanic or Latino 90.7%; other 6.8%	NR	NR	NR	NR
Thomas et al [70], 2013; United States	Prescribers: 102	NR	NR	NR	NR	NR	NR	NR	NR
Tora et al [72], 2014; Sweden	Patients: 180,059	Mean age 75.8 (SD 17.5; range 1-110) y	Female 62%	NR	NR	NR	NR	NR	NR
Watterson et al [73], 2022; United States	CS discontinuations: 49,129	NR	NR	NR	NR	NR	NR	NR	NR
Weingart et al [75], 2014; United States	Alerts: 29,592	NR	NR	NR	NR	NR	NR	NR	NR
Weingart et al [74], 2009; United States	Patients: 60,352; prescribers: 2321	NR	NR	NR	NR	NR	NR	NR	NR

^a^Only research studies are included in this table; the gray literature report is not reflected in this table owing to inability to extract the relevant information.

^b^NR: not reported.

^c^CS: controlled substance.

^d^CPOE: computerized physician order entry.

### Types of Opioids Studied

The opioids that were studied included oxycodone (14/33, 42%) [47,48,52,53,56-58,60,61,64-66,69,77], codeine (8/33, 24%) [45,48,52,57,60,61,69,77], morphine (8/33, 24%; immediate release: n=2, 25% [47,52]; controlled release: n=2, 25% [47,52]; intravenous: n=2, 25% [53,58]; and unknown: n=4, 50% [48,55,69,77]), hydromorphone (7/33, 21%) [47,48,52,57,58,64,77], tramadol (6/33, 18%) [47,48,57,69,72,77], hydrocodone (6/33, 18%) [48,52,60,65,69,77], fentanyl (5/33, 15%) [47,58,60,62,77], meperidine (3/33, 9%) [60,69,77], oxycontin (2/33, 6%) [52,54], oxymorphone (2/33, 6%) [69,77], opioid in combination with acetaminophen (hydrocodone: 3/33, 9% [56,57,60]; codeine: 2/33, 6% [57,60]; and oxycodone: 2/33, 6% [56,60]), butorphanol (1/33, 3%) [77], dihydrocodeine (1/33, 3%) [77], and tapentadol (1/33, 3%) [69].

### e-Prescribing System and Components of the System

#### Systems

The 2 main e-prescribing systems included computerized physician order entry (CPOE) prescribing (9/32, 28%) [28,46,50,55,59-61,63,65] and the electronic prescribing for controlled substances (EPCS) system (6/32, 19%) [49,51,64,67,70,71] (Table 3). Integrated into some CPOE systems, EPCS is a secure web-based system specifically for controlled substances, which allows the direct transmission of prescriptions from a prescriber to a pharmacy.

**Table 3:** e-Prescribing system and components of the system (n=32).

Study	Systems			Components							
	CPOE^a^ (n=9)	EPCS^b^ (n=6)	Dose quantity defaults and order sets (n=8)		Alerts (n=7)	Two-way communication (n=4)	Drug-drug interaction screening software (n=1)	Adherence tracking (n=1)	Computerized calculations (n=1)	Prescription printing (n=1)	Patient information (n=1)
Fischer et al [28]	✓										
Abdel-Qader et al [45]						✓					
Ancker et al [46]	✓				✓						
Bicket et al [47]											✓
Chiu et al [48]			✓								
Danovich et al [49]		✓									
Delgado Sánchez et al [50]	✓										
Everson et al [51]		✓									
George et al [52]						✓			✓	✓	
Griffey et al [53]			✓								
Hung et al [55]	✓					✓		✓			
Jones et al [56]			✓			✓					
Kearney et al [57]			✓								
Kim et al [58]			✓								
Kurteva et al [59]	✓										
Leung et al [60]	✓										
McPhillips et al [61]	✓										
Moura et al [62]					✓		✓				
Ney and Weathers [63]	✓										
Ramaseshan et al [64]		✓									
Santistevan et al [65]	✓		✓								
Schwartz et al [66]			✓								
Shoji et al [67]		✓									
Siff et al [68]					✓						
Slovis et al [69]			✓								
Thomas et al [70]		✓									
Thomas et al [71]		✓									
Tora et al [72]					✓						
Watterson et al [73]						✓					
Weingart et al [74]					✓						
Weingart et al [75]					✓						
Ariosto [77]					✓						

^a^CPOE: computerized physician order entry.

^b^EPCS: electronic prescribing for controlled substances.

#### Components

Dose quantity defaults and order sets were the most described components of the e-prescribing systems (8/32, 25%) [48,53,56-58,65,66,69] (Table 3). Alerts were the next most common component of e-prescribing software (7/32, 22%) [46,62,68,72,74,75,77]. Two-way communication between prescribers and dispensers was discussed in 5 (16%) of the 32 articles [45,52,55,56,73]. The types of communication included pharmacists reacting to a medication error and contacting medical prescribers (2/5, 40%) [45,73], medication reconciliation using enhanced computerized decision-making (ie, comparing old prescriptions and performing potential duplicate medication checks; 1/5, 20%) [55], and double validation (manual entry into the electronic medical record system twice; 1/5, 20%) [52]. Other components of e-prescribing included drug-drug interaction screening software [62], adherence tracking [55], computerized calculations [52], prescription printing [52], and the addition of patient information into the system [47].

### Effects of e-Prescribing on Opioid Use

#### Overview

The overall effects of e-prescribing on opioid use were described by 14 (42%) of the 33 articles [28,45,47,50-52,54,55,59-61,63,64,73] (Table 4). Articles examined the influence of e-prescribing on the rates of opioid prescription, discontinuation, medication adherence, and adverse drug events.

**Table 4:** Effects of e-prescribing on opioid use (n=14).

Study	Rates of prescribing (n=3)	Discontinuation (n=2)	Medication adherence (n=1)	Adverse drug events (n=1)	Prescription errors (n=8)
Fischer et al [28]			✓		
Abdel-Qader et al [45]					✓
Bicket et al [47]					✓
Delgado Sánchez et al [50]					✓
Everson et al [51]	✓				
George et al [52]					✓
Hickman et al [54]		✓			✓
Hung et al [55]					✓
Kurteva et al [59]					✓
Leung et al [60]				✓	
McPhillips et al [61]					✓
Ney and Weathers [63]	✓				
Ramaseshan et al [64]	✓				
Watterson et al [73]		✓			

#### Rates of Prescribing, Discontinuation, Medication Adherence, and Adverse Drug Events

There were mixed findings regarding opioid prescribing rates related to e-prescribing (7/14, 50%) [28,51,54,60,63,64,73]. The retrospective study by Everson et al [51] (n=459; age not reported) identified that opioids were prescribed less often from 2013 to 2018 after the introduction of e-prescribing (from 78/100, 78% people in 2013 to 43/100, 43% people in 2018). By contrast, a cross-sectional study by Ney and Weathers [63] (n=233,390; age ≥18 y) reported that the rates of primary care physician opioid prescribing increased after the implementation of CPOE (from 7.5% to 10.4% overall and from 16.4% to 20.6% for noncancer pain), with the odds of opioid prescription substantially higher in the ambulatory care visits. With respect to the opioid dose prescribed, 2 (14%) of the 14 articles reported that the quantity of opioids being prescribed decreased after the implementation of e-prescribing [51,64].

In the retrospective study by Hickman et al [54] of outpatient CPOE prescribing (n=312; age not reported), the main reason prescribers discontinued medications was due to errors in prescribing. Relatedly, Watterson et al [73] conducted a prospective cohort study (n=49,129; age not reported) to examine the impact of the CancelRx system on reducing discrepancies between the prescribing clinic’s electronic health record and the pharmacy management software. CancelRx leverages the same electronic pathway as e-prescribing but focuses on discontinuation. Using secondary data from their single academic health system and interrupted time series analyses, Watterson et al [73] reported that successful medication discontinuations increased, as defined by reduced discrepancies between clinics and pharmacies within a 72-hour period. Furthermore, Watterson et al [73] found that the time for medication discontinuation at the pharmacies decreased (eg, from weeks to same-day discontinuations) when discontinued at the prescribing clinic after the CancelRx implementation. Watterson et al [73] concluded that CancelRx improved the communication of medication discontinuations between clinics and pharmacies.

Only 1 (7%) of the 14 studies examined the rate of nonadherence for opioids when using e-prescribing, where nonadherence was defined as prescriptions not filled [28]. Fischer et al [28] conducted a retrospective study (n=280,081 patients of all ages; n=3634 prescribers) and reported that the nonadherence rate for newly prescribed opioid e-prescriptions was 23.9% of 12,625 opioid prescriptions. Of note, these authors only reported nonadherence for e-prescribing and did not compare nonadherence with no e-prescribing. Leung et al [60] found that the number of renally related preventable adverse drug events (defined as any drug-related injury owing to error at the time of order entry) decreased after the implementation of an e-prescribing system. Specific to opioids, an example of a preventable adverse drug event found related to the renal system was the oversedation from morphine [60].

#### Prescription Errors

Of the 14 articles, 8 (57%) studied the influence of e-prescribing on prescription errors [45,47,50,52,54,55,59,61]. Of these 8 articles, 4 (50%) looked at prescription errors across various drug types and found that opioids such as codeine, morphine, and oxycodone were often associated with an error [45,54,55,61]. Typical errors for opioids included discontinuation errors (ie, prescriptions were discontinued owing to erroneous prescription entry as described by physicians), transcription errors, duplicated medications, or dosing errors [50,52,54,55]. Of the 8 articles, 3 (38%) compared the opioid error rates between e-prescriptions and handwritten prescriptions [47,59,61]. Compared with handwritten prescriptions, e-prescriptions resulted in lower risk for medication errors (20.6% vs 1.2%) [59] and lower overall guideline deviations (100% of the deviations were observed in handwritten prescriptions and not computer-generated prescriptions) [47]. However, the retrospective study conducted by McPhillips et al [61] (n=1933; age not reported) reported no difference.

### Components of e-Prescribing That Influence Opioid Use

Specific components of e-prescribing were reported to influence opioid prescribing, including alerts and default order sets.

#### Alerts

Of the 32 articles, 7 (22%) described the influence of having alerts within the e-prescribing system [46,62,68,72,74,75,77]. The types of alerts included allergy alerts [77], naloxone alerts (ie, an alert is triggered to prescribe naloxone when an opioid is being prescribed) [68], drug-drug interaction alerts [62,72,74,75], and guideline-concordance alerts [46]. Drug-drug interaction alerts were reported to have prevented serious adverse drug events in the study by Weingart et al [74] but had no effect in the study by Moura et al [62]. When looking at antiemetic drugs and their interaction with opioids, prescribers in the study by Weingart et al [75] were more likely to cancel the antiemetic drug order if the alert indicated an interaction with an opioid. With respect to guideline-concordance alerts, the study by Ancker et al [46] reported that it did not influence the total number of opioid prescriptions in a 2-week interval [46]. However, there was an increase in prescriptions that aligned with the guidelines (from 12% to 31% of all prescriptions) at an academic multispecialty practice (where concordance was previously low). This increase in aligned prescriptions was not observed at a federally qualified health center (where concordance was already high). The study by Ariosto [77] identified override rates and factors that contributed to high-volume but relatively low-value drug allergy alerts with e-prescribing. A main opioid allergy alert was found to be gastrointestinal related (eg, nausea and constipation contributing to 15% of the first alerts) [77].

#### Default Order Sets

The effect of including default order sets within the e-prescribing system was described by 8 (25%) of the 32 articles [48,53,56-58,65,66,69]. Default order sets were created within the e-prescribing system such that when a prescriber indicated that they would like to prescribe an opioid, a default quantity was provided. With respect to their effect on the prescribing patterns of opioids, 6 (75%) of the 8 articles reported a reduction in the opioid dose being prescribed [48,56,57,65,66,69], and 1 (13%) also reported a reduction in the duration of treatment [69]. Although the quantity of opioids being prescribed decreased, 1 (13%) of the 8 articles reported no change in the number of opioid prescriptions per month [69]. Medication adherence after the implementation of default order sets was described by 2 (25%) of the 8 studies [48,66]. Schwartz et al [66] found that e-prescribing assisted with a reduction in the overall quantities but did not affect the proportion of patients who reported using half or less of their prescribed opioids. Specifically, 58% (n=106) of the patients reported using half or less of the medication prescribed, and 21% (n=22) of the participants did not fill their prescriptions after the implementation of the default order set. In the study by Chiu et al [48], the authors reported no influence of default order set implementation on refill rates.

At the provider level, 4 (50%) of the 8 studies explored compliance with default order set implementation [48,53,57,58]. Of these 4 studies, 1 (25%) found that there was no change in compliance with the suggested opioid doses [57], whereas 2 (50%) found that agreement with recommendations had improved after implementation [53,58]. However, Griffey et al [53] included a caveat: although overall agreement significantly improved from before the implementation, it was still considered low (36%). Deviations from recommended doses were reported by Chiu et al [48], who suggested that the type of prescriber (resident vs attending physician) and the type of procedure being performed influenced whether the default dose was altered in new prescriptions.

### Experiences and Perceptions With e-Prescribing

Of the 33 studies included in this review, 2 (6%) described clinicians’ experiences and perceptions with using e-prescribing for opioids [70,71]. Thomas et al [71] explored barriers associated with the adoption and use of EPCS using a quantitative survey (n=246; 64% response rate). When asked about their expectations of e-prescribing systems for opioids, prescribers expected this technology to improve patient management and practice efficacy [71]. However, prescribers were hesitant to use new prescribing technologies owing to their reservations with patient confidentiality or the learning curve to use e-prescribing systems [71]. In the second study by Thomas et al [70], a survey was conducted to understand the experiences of prescribers (n=102; 68% response rate) after EPCS implementation. For prescribers currently using an e-prescribing system, they indicated that it was easy to use, improved the accuracy of prescriptions, improved workflow, improved coordination, and limited the number of calls from pharmacists [70]. With respect to satisfaction with the system, age, comfort with using a computer, the number of patients per week, and the belief that the system improved patient management were associated with increased odds of being satisfied with the system [70]. Both studies described technical issues such as computer crashes, lag time between transmitting and receiving prescriptions, and pharmacist follow-up to confirm e-prescription details as barriers to using the e-prescribing system for opioids [70,71]. Two additional barriers to the implementation of EPCS were the need to keep a security token in their possession to access the system [71] and the lack of community pharmacies using the e-prescribing system [70]. No studies explored the experiences and perspectives of patients or caregivers.

### Influence of e-Prescribing Policies or Mandates

e-Prescribing mandates were associated with the reduction of both opioid prescriptions [49,67] and opioid dose [67]. The mandates were implemented in 2 states in the United States (New York and Massachusetts) [49,67].

The single report identified in the gray literature search suggested that mandatory national use of EPCS could save the US government a projected US $53 billion [76]. The cost savings were based on several factors, including reduced costs owing to opioid-related fatalities (between US $18 billion and US $37 billion saved); decreased health care costs, including treatment costs; increase in workplace productivity; reduced criminal justice costs (between US $7 billion and US $14 billion saved); and savings from improved efficiencies in physician offices and pharmacies (eg, reduced calls between prescribers and pharmacists regarding prescription clarifications and decreased wait times for patients to fill prescriptions; US $1.6 billion saved) [76].

## Discussion

### Summary of Findings

This rapid scoping review examined how e-prescribing has been used clinically for opioids; investigated the impact on experiences, and outcomes; and identified several gaps in the literature. Overall, we identified a limited number of articles that met our inclusion criteria (n=34). Despite a comprehensive search, we identified minimal research examining e-prescribing for opioids and related outcomes. Although the results showed promising findings, such as a reduction in prescription errors and identifying drug-drug interactions, there remain important clinical, implementation, effectiveness, and policy-relevant areas for further exploration.

Most studies examined e-prescribing being initiated within hospital-based care or an affiliated ambulatory clinic. Thus, most of the evidence found in this review reflects hospital settings and closed health systems. The main data systems used within the hospital systems were the CPOE system and EPCS. Only 1 (3%) of the 34 articles focused on e-prescribing in primary care, using the CPOE system [63]. In addition, there was minimal reporting of prescriber and pharmacist characteristics, clinical characteristics, or sociodemographic information. Furthermore, we identified a large variation across the included studies examining the effects of e-prescribing on experiences, and outcomes. Most of the outcomes were focused on prescription-level metrics such as prescription rates, prescription errors, and discontinuation rates.

Despite the variation, there seem to be promising findings with respect to e-prescribing; for example, 1 (3%) of the 34 studies showed a reduction in prescribing errors when compared with handwritten notes (eg, 20.6% handwritten errors vs 1.2% e-prescription errors) [59]. A few studies (2/34, 6%) also highlighted promising effects of alerts and order sets on reducing errors; 2 (6%) of the 34 studies demonstrated the usefulness of e-prescribing mandates in reducing opioid prescriptions [49,67] and reducing dose [67]. Given the increasing rates of opioid-related harms in North America [1], these findings suggest that e-prescribing may be a promising approach to address prescribing errors. However, it is important to understand the nature and related implications of reducing the number, dose, and rapid discontinuations because there may be unintended risks of reducing access to opioids or reducing doses too quickly [78-80].

In the single gray literature report identified, the mandatory national use of EPCS has been projected to have a potential cost savings of approximately US $53 billion annually for the US government [76]. Despite uncertainty around cost savings, there is potential for these cost savings to be reallocated to fund educational programs for prescribers, patients, and the public. However, it is important to note that the unregulated opioid drug supply is the main cause of opioid-related deaths in Ontario, and the generalizability of this review to the Canadian context should be made with caution [12].

One of the challenges in reviewing the literature is the substantial shift in practice guidelines for opioid therapy management that occurred in North America after 2016 [14,15]. As such, studies published before this date examining e-prescribing and opioid use may not reflect current practices or needs. This review identified several gaps, particularly related to implementation and effectiveness considerations. Future research is warranted to expand the current knowledge of e-prescribing systems and opioid-related outcomes. First, e-prescribing needs to be assessed across broader health systems and larger populations, such as in community and primary care. Only a single study was found that assessed e-prescribing in primary care [63]. This study included data collected before 2016, when significant practice guideline changes were released that have an impact on opioid prescribing and patterns, suggesting the data only available from primary care likely do not reflect current practice or needs. Second, the perspectives, experiences, and health care outcomes from a wide variety of stakeholders (such as prescribers, clinicians, pharmacists, patients, and pharmacy managers) should be explored and examined through mixed methods and qualitative studies; for example, qualitative studies with community stakeholders would provide insight into the fear regarding the e-prescribing of opioids that has previously been reported to affect the prescribing rates of primary care physicians [81-84]. Third, the development of a common set of quality indicators to guide the reporting of outcomes would likely be useful to ensure the consistent implementation and evaluation of e-prescribing across varying studies. Finally, more studies are needed to understand implementation considerations such as barriers and facilitators for e-prescribing to inform adoption and larger scalability. There are well-established factors that influence the implementation of interventions and their effectiveness; for example, the Consolidated Framework for Implementation Research consists of 5 key domains that are known to influence implementation [85]. To inform the adoption and uptake efforts of e-prescribing technology, it will be important for future work to understand for whom e-prescribing might be working, how, and in what circumstances, which may be completed through a realist evaluation [86]. This review identified important questions that remain, such as the following: (1) Are there certain oppressed groups where this technology might be particularly useful to support safe and effective opioid therapy management? (2) Are there certain prescribers and pharmacists who might benefit more from this technology and in what clinical settings? (3) Are there certain aspects of the e-prescribing system that are more beneficial or harmful (eg, questions related to the alerts, order sets, and interaction features)? Of note, there are known risks to rapid dose reductions with opioids [78], and it would be important to explore further an understanding of potential harms. A key aspect for consideration is how e-prescribing might be implemented for new prescriptions to prevent short- and long-term risks among persons compared with how it might be implemented for repeat prescriptions among those experiencing chronic pain. These implementation factors should be considered in future work examining e-prescribing.

Overall, there was a lack of consistency in the types of outcomes reported, and it is unclear whether the outcomes reported align with established quality indicators (eg, a consideration of dose within the clinical context of acute or chronic care). Several of the outcomes may be problematic, such as nonadherence and discontinuation, because they may not accurately reflect an improvement in outcomes; for example, with nonadherence, it is important to consider differences in “taking medication when needed” versus “taking medication on a prescribed schedule.” With respect to discontinuation, the timing needs to be considered (eg, discontinuing the same day vs discontinuing within the prescription period). Same-day discontinuations are likely due to errors by the prescriber, as seen in the study conducted by Hickman et al [54]. Tapering guidelines for chronic pain suggest that the discontinuation of opioids may lead to the risk of inadvertent or unintentional overdose risk, if not carried out properly [87,88]. It is suggested that patients follow a gradual morphine equivalent dose decrease of 5% to 10 % every 2 to 4 weeks with frequent follow-up. However, if the prescription is for acute pain, tapering is not necessarily needed [87,88]. Finally, there was an absence of studies exploring the perceptions of e-prescribing for opioids from different stakeholder groups (eg, clinicians, prescribers, and patients) from a qualitative perspective, which would also inform meaningful outcomes and potential indicators of quality e-prescribing.

The limitations of this study are consistent with those common to rapid reviews. It is possible that articles were missed. Despite the time constraint, a rigorous selection process was undertaken with double screening present at each stage of the process, and grey literature was searched. Of note, 15 (44%) of the 34 articles were published in 2016 or earlier, which would not reflect the dramatic shifts that occurred in opioid therapy management in the last several years. In addition, the quality of the studies was not assessed, which is typical of a scoping review, and as such, this review does not integrate the strength of the evidence [35].

### Conclusions

Although relatively few studies were identified, this scoping review highlights preliminarily promising results with e-prescribing and opioid therapy management. e-Prescribing, including key features such as alerts and dose order sets, may contribute to a reduction in prescribing errors. A key aspect for consideration is how e-prescribing might be used and the differences in outcomes by using this tool based on medication prescription being newly initiated or chronic. Among *new prescriptions,* there may be potential to decrease initiation, quantities, and doses as per best practice guidelines to minimize short- and long-term risks. Conversely, there may be important and different considerations with e-prescribing for people who are taking opioids on a *chronic basis* to minimize disruptions with access and sudden dose changes. These important nuances were missed from the research reviewed and highlight gaps in the literature. It is important that future work explores the experience of prescribers, pharmacists, and patients using e-prescribing for opioid therapy management, with an emphasis on prescribers in the community and primary care. Integrating the thoughts, perceptions, and beliefs of these parties into the literature is important because they are directly affected by technology use in health care. Developing a common set of quality indicators for e-prescribing with opioids will help inform future research and build a stronger evidence base. Furthermore, understanding implementation considerations will be required as the technology is adopted and integrated into clinical practice and health systems.

## Appendix

Multimedia Appendix 1 PRISMA-ScR (Preferred Reporting Items for Systematic Reviews and Meta-Analyses extension for Scoping Reviews) checklist.

Multimedia Appendix 2 Search strategies for the databases and grey literature.

## Abbreviations

CPOE: computerized physician order entry
EPCS: electronic prescribing for controlled substances
PRESS: Peer Review of Electronic Search Strategies
PRISMA: Preferred Reporting Items for Systematic Reviews and Meta-Analyses
PRISMA-ScR: Preferred Reporting Items for Systematic Reviews and Meta-Analyses extension for Scoping Reviews
