# Supplementary material for: Experiences and Outcomes of Using e-Prescribing for Opioids: Rapid Scoping Review
Source: J Med Internet Res. 2023 Dec 28;25:e49173. doi: 10.2196/49173 (PMC10784986; doi:10.2196/49173)
Supplement: Multimedia Appendix 2 [file jmir_v25i1e49173_app2.docx]

|  | **MEDLINE All (Ovid)** | **Results** |
| --- | --- | --- |
| 1 | Drug Therapy, Computer-Assisted/ | 1691 |
| 2 | Electronic Prescribing/ | 1162 |
| 3 | Medical Order Entry Systems/ | 2439 |
| 4 | (eprescrib* or eprescription*).ti,ab,kw. | 106 |
| 5 | (e prescrib* or e prescription*).ti,ab,kw. | 573 |
| 6 | (epharmacy or epharmacies).ti,ab,kw. | 0 |
| 7 | (e pharmacy or e pharmacies).ti,ab,kw. | 29 |
| 8 | ((computeri?ed or digital* or electronic* or internet or online or virtual*) adj2 (prescrib* or prescription*)).ti,ab,kw. | 2311 |
| 9 | ((computeri?ed or digital* or electronic* or internet or online or virtual*) adj2 (pharmacy or pharmacies)).ti,ab,kw. | 916 |
| 10 | (electronic* adj2 (transmit* or transmission or send* or sent) adj2 (prescrib* or prescription*)).ti,ab,kw. | 55 |
| 11 | (e medicine management or emedicine management).ti,ab,kw. | 0 |
| 12 | (computer* adj3 (entry system* or order system* or order entry or order management or drug therap*)).ti,ab,kw. | 1911 |
| 13 | (pharmac* management system* or order entry system* or order management system*).ti,ab,kw. | 747 |
| 14 | ((pharmac* or prescrib* or prescription*) adj3 software*).ti,ab,kw. | 696 |
| 15 | or/1-14 [e-prescribing] | 9008 |
| 16 | exp Narcotics/ | 140632 |
| 17 | exp Opioid-Related Disorders/ | 32948 |
| 18 | Alphaprodine/ or exp Buprenorphine/ or Butorphanol/ or exp Codeine/ or Dextromoramide/ or Dextropropoxyphene/ or exp Enkephalins/ or exp Fentanyl/ or Hydromorphone/ or Levorphanol/ or exp Meperidine/ or Meptazinol/ or exp Methadone/ or Morphine/ or Nalbuphine/ or Oxymorphone/ or Pentazocine/ or Phenoperidine/ or Pirinitramide/ or Tramadol/ | 105016 |
| 19 | (opioid* or opiate* or narcotic*).ti,ab,kw. | 143111 |
| 20 | (alphaprodine or nisentil or prodine).ti,ab,kw. | 147 |
| 21 | (buprenorphine or buprenorphine or buprenex or buprex or prefin or subutex or temgesic or 6029 m or 6029m or rx6029m or suboxone).ti,ab,kw. | 8859 |
| 22 | (butorphanol or dolorex or moradol or stadol or torbugesic or bc 2627 or bc2627).ti,ab,kw. | 1733 |
| 23 | (codeine or ardinex or idocodeine or n methylmorphine or hydrocodone or codinovo or dicodid or dihydrocodeinone or hycodan or hycon or hydrocodeinonebitartrate or robidone or oxycodone or dihydrohydroxycodeinone or dihydrone or dinarkon or eucodal or oxiconum or oxycodeinon or oxycone or oxycontin or pancodine or percocet or theocodin or vicodin).ti,ab,kw. | 10300 |
| 24 | (dextromoramide or d moramide or palfium or pyrrolamidol).ti,ab,kw. | 272 |
| 25 | (dextropropoxyphene or propoxyphene or d propoxyphene or darvon).ti,ab,kw. | 1474 |
| 26 | (enkephalin* or dago or dagol or damge or damgo or rx 783006 or dpdpe).ti,ab,kw. | 17709 |
| 27 | (fentanyl or duragesic or durogesic or fentanest or fentora or phentanyl or r 4263 or r4263 or sublimaze or alfenta or alfentanil or fanaxal or limifen or rapifen or r 39209 or r39209 or sufentanil* or sulfentanil or sulfentanyl or r 30730 or r30730).ti,ab,kw. | 25228 |
| 28 | (hydromorphon* or dihydromorphinone or dilaudid or laudacon or palladone).ti,ab,kw. | 2016 |
| 29 | (levorphanol or levodroman or levorphan or levo dromoran or l dromoran).ti,ab,kw. | 490 |
| 30 | medical heroin.ti,ab,kw. | 7 |
| 31 | (meperidine or demerol or dolantin or dolargan or dolcontral or dolin or dolosal or dolsin or isonipecain or lidol or lydol or operidine or pethidine or promedol or dimethylmeperidine or isopromedol or trimeperidine).ti,ab,kw. | 6032 |
| 32 | (meptazinol or meptid or wy 22811 or wy22811).ti,ab,kw. | 227 |
| 33 | (methadone or biodone or dolophine or metadol or metasedin or symoron or methadose or methex or phenadone or physeptone or phymet or pinadone or amidone or methaddict or methadyl or acetylmethadol or alphacetylmethadol or dimepheptanol or levomethadyl or levoacetylmethadol or laam or methadol or orlaam or acemethadone).ti,ab,kw. | 15560 |
| 34 | (morphin* or morphia or duramorph or ms contin or morphia or oramorph sr or sdz 202 250 or sdz202250 or sdz202 250).ti,ab,kw. | 58262 |
| 35 | (nalbuphine or nubain* or en 2234a or en2234a).ti,ab,kw. | 1087 |
| 36 | (oxymorphone or numorphan or opana).ti,ab,kw. | 682 |
| 37 | (pentazocine or fortral or lexir or talwin).ti,ab,kw. | 2462 |
| 38 | (phenoperidine or fenoperidine or lealgin or operidine or r 1406 or r1406).ti,ab,kw. | 157 |
| 39 | (pirinitramid* or piritramid* or dipidolor or dipydolor).ti,ab,kw. | 475 |
| 40 | (tramadol or adolonta or amadol or biodalgic or biokanol or contramal or jutadol or k 315 or k315 or mtwtramadol or nobligan or prontofort or ranitidin 1a pharma or takadol or theradol or tiral or topalgic or tradol or tradolpuren or tradonal or tralgiol or trama or tramadorsch or tramabeta or tramadin or tramadoc or tramadoldolgit or tramadolhameln or tramadolor or tramadolratiopharm or tramadura or tramagetic or tramagit or tramake or tramal or tramex or tramundin or trasedal or ultram or xymel 50 or zamudol or zumalgic or zydol or zytram).ti,ab,kw. | 6340 |
| 41 | (acetorophine or acetylcodeine or acetymethadol or anileridine or apadoline or azidomorphine or benzhydrocodone or bezitramide or bremazocine or brompton mixture or ciramadol or cocomadol or codydramol or conorfone or cyclazocine or dextrorphan or dezocine or diamorphine or diconal or dihydroetorphine or dihydromorphine or dimethylthiambutene or dipipanone or dynorphin or enadoline or eptazocine or ethylketazocine or ethylmorphine or etonitazene or etorphine or etoxeridine or faxeladol or furethidine or gelonida or isalmadol or isomethodone or ketazocine or ketobemidone or ketogan or kyotorphin or lefetamine or levacetylmethadol or levomethadone or levorphanol or metazocine or methylsamidorphan or tilidine or nicodine or nicomorphine or noracymethadol or bufigen pentor nalbufin* or nalcryn or nalpain or onfor or noracymethadol or norbuprenorphine or normorphine or norpethidine or norpropoxyphene or nortramadol or oliceridine or oripavine or pentamorphone or phenadoxone or phencyclidine or picenadol or piminodine or piritramide or profadol or propiram or sameridine or samidorphan or semorphone or tapentadol or thebaine or tifluadom or tilidine or tonazocine).ti,ab,kw. | 16508 |
| 42 | or/16-41 [opioids] | 259653 |
| **43** | **15 and 42** | **353** |

|  | **Embase (Elsevier Embase.com)** | **Results** |
| --- | --- | --- |
| 1 | 'computer assisted drug therapy'/de | 931 |
| 2 | 'computerized provider order entry'/exp | 6194 |
| 3 | 'physician order entry system'/de | 340 |
| 4 | (eprescrib* OR eprescription*):ti,ab,kw | 1195 |
| 5 | ('e prescrib*' OR 'e prescription*'):ti,ab,kw | 1192 |
| 6 | (epharmacy OR epharmacies):ti,ab,kw | 64 |
| 7 | ('e pharmacy' OR 'e pharmacies'):ti,ab,kw | 88 |
| 8 | ((computeri?ed OR digital* OR electronic* OR internet OR online OR virtual*) NEAR/2 (prescrib* OR prescription*)):ti,ab,kw | 4675 |
| 9 | ((computeri?ed OR digital* OR electronic* OR internet OR online OR virtual*) NEAR/2 (pharmacy OR pharmacies)):ti,ab,kw | 1633 |
| 10 | (electronic* NEAR/2 (transmit* OR transmission OR send* OR sent) NEAR/2 (prescrib* OR prescription*)):ti,ab,kw | 93 |
| 11 | ('e medicine management' OR 'emedicine management'):ti,ab,kw | 0 |
| 12 | (computer* NEAR/3 ('entry system*' OR 'order system*' OR 'order entry' OR 'order management' OR 'drug therap*')):ti,ab,kw | 2951 |
| 13 | ('pharmac* management system*' OR 'order entry system*' OR 'order management system*'):ti,ab,kw | 1291 |
| 14 | ((pharmac* OR prescrib* OR prescription*) NEAR/3 software*):ti,ab,kw | 1607 |
| 15 | #1 OR #2 OR #3 OR #4 OR #5 OR #6 OR #7 OR #8 OR #9 OR #10 OR #11 OR #12 OR #13 OR #14 | 14275 |
| 16 | 'narcotic agent'/de OR 'narcotic analgesic agent'/de | 42025 |
| 17 | 'opiate agonist'/de | 6504 |
| 18 | 'acetylmethadol'/de OR 'alfentanil'/de OR 'alphaprodine'/de OR 'buprenorphine'/de OR 'buprenorphine plus naloxone'/de OR 'butorphanol'/de OR 'codeine'/de OR 'dextromoramide'/de OR 'dextropropoxyphene'/de OR 'enkephalin'/exp OR 'fentanyl'/de OR 'hydrocodone'/de OR 'hydromorphone'/de OR 'levorphanol'/de OR 'meptazinol'/de OR 'methadone'/de OR 'morphine'/de OR 'nalbuphine'/exp OR 'oxycodone'/de OR 'oxymorphone'/de OR 'pethidine'/de OR 'pentazocine'/de OR 'phenoperidine'/de OR 'piritramide'/de OR 'sufentanil'/de OR 'tramadol'/de OR 'trimeperidine'/de | 298233 |
| 19 | (opioid* OR opiate* OR narcotic*):ti,ab,kw | 201063 |
| 20 | (alphaprodine OR nisentil OR prodine):ti,ab,kw | 148 |
| 21 | (buprenorphine OR buprenorphine OR buprenex OR buprex OR prefin OR subutex OR temgesic OR '6029 m' OR 6029m OR rx6029m OR suboxone):ti,ab,kw | 12885 |
| 22 | (butorphanol OR dolorex OR moradol OR stadol OR torbugesic OR 'bc 2627' OR bc2627):ti,ab,kw | 2121 |
| 23 | (codeine OR ardinex OR idocodeine OR 'n methylmorphine' OR hydrocodone OR codinovo OR dicodid OR dihydrocodeinone OR hycodan OR hycon OR hydrocodeinonebitartrate OR robidone OR oxycodone OR dihydrohydroxycodeinone OR dihydrone OR dinarkon OR eucodal OR oxiconum OR oxycodeinon OR oxycone OR oxycontin OR pancodine OR percocet OR theocodin OR vicodin):ti,ab,kw | 16490 |
| 24 | (dextromoramide OR 'd moramide' OR palfium OR pyrrolamidol):ti,ab,kw | 262 |
| 25 | (dextropropoxyphene OR propoxyphene OR 'd propoxyphene' OR darvon):ti,ab,kw | 1931 |
| 26 | (enkephalin* OR dago OR dagol OR damge OR damgo OR 'rx 783006' OR dpdpe):ti,ab,kw | 20278 |
| 27 | (fentanyl OR duragesic OR durogesic OR fentanest OR fentora OR phentanyl OR 'r 4263' OR r4263 OR sublimaze OR alfenta OR alfentanil OR fanaxal OR limifen OR rapifen OR 'r 39209' OR r39209 OR sufentanil* OR sulfentanil OR sulfentanyl OR 'r 30730' OR r30730):ti,ab,kw | 37218 |
| 28 | (hydromorphon* OR dihydromorphinone OR dilaudid OR laudacon OR palladone):ti,ab,kw | 3570 |
| 29 | (levorphanol OR levodroman OR levorphan OR 'levo dromoran' OR 'l dromoran'):ti,ab,kw | 606 |
| 30 | 'medical heroin':ti,ab,kw | 12 |
| 31 | (meperidine OR demerol OR dolantin OR dolargan OR dolcontral OR dolin OR dolosal OR dolsin OR isonipecain OR lidol OR lydol OR operidine OR pethidine OR promedol OR dimethylmeperidine OR isopromedol OR trimeperidine):ti,ab,kw | 7643 |
| 32 | (meptazinol OR meptid OR 'wy 22811' OR wy22811):ti,ab,kw | 267 |
| 33 | (methadone OR biodone OR dolophine OR metadol OR metasedin OR symoron OR methadose OR methex OR phenadone OR physeptone OR phymet OR pinadone OR amidone OR methaddict OR methadyl OR acetylmethadol OR alphacetylmethadol OR dimepheptanol OR levomethadyl OR levoacetylmethadol OR laam OR methadol OR orlaam OR acemethadone):ti,ab,kw | 21687 |
| 34 | (morphin* OR morphia OR duramorph OR 'ms contin' OR morphia OR 'oramorph sr' OR 'sdz 202 250' OR sdz202250 OR 'sdz202 250'):ti,ab,kw | 78039 |
| 35 | (nalbuphine OR nubain* OR 'en 2234a' OR en2234a):ti,ab,kw | 1422 |
| 36 | (oxymorphone OR numorphan OR opana):ti,ab,kw | 958 |
| 37 | (pentazocine OR fortral OR lexir OR talwin):ti,ab,kw | 3330 |
| 38 | (phenoperidine OR fenoperidine OR lealgin OR operidine OR 'r 1406' OR r1406):ti,ab,kw | 178 |
| 39 | (pirinitramid* OR piritramid* OR dipidolor OR dipydolor):ti,ab,kw | 622 |
| 40 | (tramadol OR adolonta OR amadol OR biodalgic OR biokanol OR contramal OR jutadol OR 'k 315' OR k315 OR mtwtramadol OR nobligan OR prontofort OR 'ranitidin 1a pharma5' OR takadol OR theradol OR tiral OR topalgic OR tradol OR tradolpuren OR tradonal OR tralgiol OR trama OR tramadorsch OR tramabeta OR tramadin OR tramadoc OR tramadoldolgit OR tramadolhameln OR tramadolor OR tramadolratiopharm OR tramadura OR tramagetic OR tramagit OR tramake OR tramal OR tramex OR tramundin OR trasedal OR ultram OR 'xymel 50' OR zamudol OR zumalgic OR zydol OR zytram):ti,ab,kw | 10737 |
| 41 | (acetorophine OR acetylcodeine OR acetymethadol OR anileridine OR apadoline OR azidomorphine OR benzhydrocodone OR bezitramide OR bremazocine OR 'brompton mixture' OR ciramadol OR cocomadol OR codydramol OR conorfone OR cyclazocine OR dextrorphan OR dezocine OR diamorphine OR diconal OR dihydroetorphine OR dihydromorphine OR dimethylthiambutene OR dipipanone OR dynorphin OR enadoline OR eptazocine OR ethylketazocine OR ethylmorphine OR etonitazene OR etorphine OR etoxeridine OR faxeladol OR furethidine OR gelonida OR isalmadol OR isomethodone OR ketazocine OR ketobemidone OR ketogan OR kyotorphin OR lefetamine OR levacetylmethadol OR levomethadone OR levorphanol OR metazocine OR methylsamidorphan OR tilidine OR nicodine OR nicomorphine OR noracymethadol OR 'bufigen pentor nalbufin*' OR nalcryn OR nalpain OR onfor OR noracymethadol OR norbuprenorphine OR normorphine OR norpethidine OR norpropoxyphene OR nortramadol OR oliceridine OR oripavine OR pentamorphone OR phenadoxone OR phencyclidine OR picenadol OR piminodine OR piritramide OR profadol OR propiram OR sameridine OR samidorphan OR semorphone OR tapentadol OR thebaine OR tifluadom OR tilidine OR tonazocine):ti,ab,kw | 21063 |
| 42 | #16 OR #17 OR #18 OR #19 OR #20 OR #21 OR #22 OR #23 OR #24 OR #25 OR #26 OR #27 OR #28 OR #29 OR #30 OR #31 OR #32 OR #33 OR #34 OR #35 OR #36 OR #37 OR #38 OR #39 OR #40 OR #41 | 445721 |
| 43 | **#15 AND #42** | **799** |

|  | **Scopus (Elsevier Scopus.com)** | **Results** |
| --- | --- | --- |
| 1 | (TITLE-ABS-KEY(((computeri?ed OR digital* OR electronic* OR internet OR online OR virtual*) W/2 (prescrib* OR prescription* OR pharmacy OR pharmacies)) OR (electronic* W/2 (transmit* OR transmission OR send* OR sent) W/2 (prescrib* OR prescription*)) OR "e medicine management" OR "emedicine management" OR (computer* W/3 ("entry system*" OR "order system*" OR "order entry" OR "order management" OR "drug therap*")) OR "pharmac* management system*" OR "order entry system*" OR "order management system*" OR ((pharmac* OR prescrib* OR prescription*) W/3 software*))) AND NOT ((INDEX(medline)) OR (INDEX(embase))) | 2182 |
| 2 | (TITLE-ABS-KEY(opioid* OR opiate* OR narcotic* OR alphaprodine OR nisentil OR prodine OR buprenorphine OR buprenorphine OR buprenex OR buprex OR prefin OR subutex OR temgesic OR "6029 m" OR 6029m OR rx6029m OR suboxone OR butorphanol OR dolorex OR moradol OR stadol OR torbugesic OR "bc 2627" OR bc2627 OR codeine OR ardinex OR idocodeine OR "n methylmorphine" OR hydrocodone OR codinovo OR dicodid OR dihydrocodeinone OR hycodan OR hycon OR hydrocodeinonebitartrate OR robidone OR oxycodone OR dihydrohydroxycodeinone OR dihydrone OR dinarkon OR eucodal OR oxiconum OR oxycodeinon OR oxycone OR oxycontin OR pancodine OR percocet OR theocodin OR vicodin OR dextromoramide OR "d moramide" OR palfium OR pyrrolamidol OR dextropropoxyphene OR propoxyphene OR "d propoxyphene" OR darvon OR enkephalin* OR dago OR dagol OR damge OR damgo OR "rx 783006" OR dpdpe OR fentanyl OR duragesic OR durogesic OR fentanest OR fentora OR phentanyl OR "r 4263" OR r4263 OR sublimaze OR alfenta OR alfentanil OR fanaxal OR limifen OR rapifen OR "r 39209" OR r39209 OR sufentanil* OR sulfentanil OR sulfentanyl OR "r 30730" OR r30730 OR hydromorphon* OR dihydromorphinone OR dilaudid OR laudacon OR palladone OR levorphanol OR levodroman OR levorphan OR "levo dromoran" OR "l dromoran" OR "medical heroin" OR meperidine OR demerol OR dolantin OR dolargan OR dolcontral OR dolin OR dolosal OR dolsin OR isonipecain OR lidol OR lydol OR operidine OR pethidine OR promedol OR dimethylmeperidine OR isopromedol OR trimeperidine OR meptazinol OR meptid OR "wy 22811" OR wy22811 OR methadone OR biodone OR dolophine OR metadol OR metasedin OR symoron OR methadose OR methex OR phenadone OR physeptone OR phymet OR pinadone OR amidone OR methaddict OR methadyl OR acetylmethadol OR alphacetylmethadol OR dimepheptanol OR levomethadyl OR levoacetylmethadol OR laam OR methadol OR orlaam OR acemethadone OR morphin* OR morphia OR duramorph OR "ms contin" OR morphia OR "oramorph sr" OR "sdz 202 250" OR sdz202250 OR "sdz202 250" OR nalbuphine OR nubain* OR "en 2234a" OR en2234a OR oxymorphone OR numorphan OR opana OR pentazocine OR fortral OR lexir OR talwin OR phenoperidine OR fenoperidine OR lealgin OR operidine OR "r 1406" OR r1406 OR pirinitramid* OR piritramid* OR dipidolor OR dipydolor OR tramadol OR adolonta OR amadol OR biodalgic OR biokanol OR contramal OR jutadol OR "k 315" OR k315 OR mtwtramadol OR nobligan OR prontofort OR "ranitidin 1a pharma5" OR takadol OR theradol OR tiral OR topalgic OR tradol OR tradolpuren OR tradonal OR tralgiol OR trama OR tramadorsch OR tramabeta OR tramadin OR tramadoc OR tramadoldolgit OR tramadolhameln OR tramadolor OR tramadolratiopharm OR tramadura OR tramagetic OR tramagit OR tramake OR tramal OR tramex OR tramundin OR trasedal OR ultram OR "xymel 50" OR zamudol OR zumalgic OR zydol OR zytram OR acetorophine OR acetylcodeine OR acetymethadol OR anileridine OR apadoline OR azidomorphine OR benzhydrocodone OR bezitramide OR bremazocine OR "brompton mixture" OR ciramadol OR cocomadol OR codydramol OR conorfone OR cyclazocine OR dextrorphan OR dezocine OR diamorphine OR diconal OR dihydroetorphine OR dihydromorphine OR dimethylthiambutene OR dipipanone OR dynorphin OR enadoline OR eptazocine OR ethylketazocine OR ethylmorphine OR etonitazene OR etorphine OR etoxeridine OR faxeladol OR furethidine OR gelonida OR isalmadol OR isomethodone OR ketazocine OR ketobemidone OR ketogan OR kyotorphin OR lefetamine OR levacetylmethadol OR levomethadone OR levorphanol OR metazocine OR methylsamidorphan OR tilidine OR nicodine OR nicomorphine OR noracymethadol OR "bufigen pentor nalbufin*" OR nalcryn OR nalpain OR onfor OR noracymethadol OR norbuprenorphine OR normorphine OR norpethidine OR norpropoxyphene OR nortramadol OR oliceridine OR oripavine OR pentamorphone OR phenadoxone OR phencyclidine OR picenadol OR piminodine OR piritramide OR profadol OR propiram OR sameridine OR samidorphan OR semorphone OR tapentadol OR thebaine OR tifluadom OR tilidine OR tonazocine)) AND NOT ((INDEX(medline)) OR (INDEX(embase))) | 38287 |
| **3** | **#1 and #2 *[using search history tool]*** | **31** |

|  | **Dissertations & Theses Global (ProQuest)** | **Results** |
| --- | --- | --- |
| 1 | TITLE(((computeri?ed OR digital* OR electronic* OR internet OR online OR virtual*) N/2 (prescrib* OR prescription* OR pharmacy OR pharmacies)) OR (electronic* N/2 (transmit* OR transmission OR send* OR sent) N/2 (prescrib* OR prescription*)) OR "e medicine management" OR "emedicine management" OR (computer* N/3 ("entry system*" OR "order system*" OR "order entry" OR "order management" OR "drug therap*")) OR "pharmac* management system*" OR "order entry system*" OR "order management system*" OR ((pharmac* OR prescrib* OR prescription*) N/3 software*)) OR ABSTRACT(((computeri?ed OR digital* OR electronic* OR internet OR online OR virtual*) N/2 (prescrib* OR prescription* OR pharmacy OR pharmacies)) OR (electronic* N/2 (transmit* OR transmission OR send* OR sent) N/2 (prescrib* OR prescription*)) OR "e medicine management" OR "emedicine management" OR (computer* N/3 ("entry system*" OR "order system*" OR "order entry" OR "order management" OR "drug therap*")) OR "pharmac* management system*" OR "order entry system*" OR "order management system*" OR ((pharmac* OR prescrib* OR prescription*) N/3 software*)) | 391 |
| 2 | TITLE(opioid* OR opiate* OR narcotic* OR alphaprodine OR nisentil OR prodine OR buprenorphine OR buprenorphine OR buprenex OR buprex OR prefin OR subutex OR temgesic OR "6029 m" OR 6029m OR rx6029m OR suboxone OR butorphanol OR dolorex OR moradol OR stadol OR torbugesic OR "bc 2627" OR bc2627 OR codeine OR ardinex OR idocodeine OR "n methylmorphine" OR hydrocodone OR codinovo OR dicodid OR dihydrocodeinone OR hycodan OR hycon OR hydrocodeinonebitartrate OR robidone OR oxycodone OR dihydrohydroxycodeinone OR dihydrone OR dinarkon OR eucodal OR oxiconum OR oxycodeinon OR oxycone OR oxycontin OR pancodine OR percocet OR theocodin OR vicodin OR dextromoramide OR "d moramide" OR palfium OR pyrrolamidol OR dextropropoxyphene OR propoxyphene OR "d propoxyphene" OR darvon OR enkephalin* OR dago OR dagol OR damge OR damgo OR "rx 783006" OR dpdpe OR fentanyl OR duragesic OR durogesic OR fentanest OR fentora OR phentanyl OR "r 4263" OR r4263 OR sublimaze OR alfenta OR alfentanil OR fanaxal OR limifen OR rapifen OR "r 39209" OR r39209 OR sufentanil* OR sulfentanil OR sulfentanyl OR "r 30730" OR r30730 OR hydromorphon* OR dihydromorphinone OR dilaudid OR laudacon OR palladone OR levorphanol OR levodroman OR levorphan OR "levo dromoran" OR "l dromoran" OR "medical heroin" OR meperidine OR demerol OR dolantin OR dolargan OR dolcontral OR dolin OR dolosal OR dolsin OR isonipecain OR lidol OR lydol OR operidine OR pethidine OR promedol OR dimethylmeperidine OR isopromedol OR trimeperidine OR meptazinol OR meptid OR "wy 22811" OR wy22811 OR methadone OR biodone OR dolophine OR metadol OR metasedin OR symoron OR methadose OR methex OR phenadone OR physeptone OR phymet OR pinadone OR amidone OR methaddict OR methadyl OR acetylmethadol OR alphacetylmethadol OR dimepheptanol OR levomethadyl OR levoacetylmethadol OR laam OR methadol OR orlaam OR acemethadone OR morphin* OR morphia OR duramorph OR "ms contin" OR morphia OR "oramorph sr" OR "sdz 202 250" OR sdz202250 OR "sdz202 250" OR nalbuphine OR nubain* OR "en 2234a" OR en2234a OR oxymorphone OR numorphan OR opana OR pentazocine OR fortral OR lexir OR talwin OR phenoperidine OR fenoperidine OR lealgin OR operidine OR "r 1406" OR r1406 OR pirinitramid* OR piritramid* OR dipidolor OR dipydolor OR tramadol OR adolonta OR amadol OR biodalgic OR biokanol OR contramal OR jutadol OR "k 315" OR k315 OR mtwtramadol OR nobligan OR prontofort OR "ranitidin 1a pharma5" OR takadol OR theradol OR tiral OR topalgic OR tradol OR tradolpuren OR tradonal OR tralgiol OR trama OR tramadorsch OR tramabeta OR tramadin OR tramadoc OR tramadoldolgit OR tramadolhameln OR tramadolor OR tramadolratiopharm OR tramadura OR tramagetic OR tramagit OR tramake OR tramal OR tramex OR tramundin OR trasedal OR ultram OR "xymel 50" OR zamudol OR zumalgic OR zydol OR zytram OR acetorophine OR acetylcodeine OR acetymethadol OR anileridine OR apadoline OR azidomorphine OR benzhydrocodone OR bezitramide OR bremazocine OR "brompton mixture" OR ciramadol OR cocomadol OR codydramol OR conorfone OR cyclazocine OR dextrorphan OR dezocine OR diamorphine OR diconal OR dihydroetorphine OR dihydromorphine OR dimethylthiambutene OR dipipanone OR dynorphin OR enadoline OR eptazocine OR ethylketazocine OR ethylmorphine OR etonitazene OR etorphine OR etoxeridine OR faxeladol OR furethidine OR gelonida OR isalmadol OR isomethodone OR ketazocine OR ketobemidone OR ketogan OR kyotorphin OR lefetamine OR levacetylmethadol OR levomethadone OR levorphanol OR metazocine OR methylsamidorphan OR tilidine OR nicodine OR nicomorphine OR noracymethadol OR "bufigen pentor nalbufin*" OR nalcryn OR nalpain OR onfor OR noracymethadol OR norbuprenorphine OR normorphine OR norpethidine OR norpropoxyphene OR nortramadol OR oliceridine OR oripavine OR pentamorphone OR phenadoxone OR phencyclidine OR picenadol OR piminodine OR piritramide OR profadol OR propiram OR sameridine OR samidorphan OR semorphone OR tapentadol OR thebaine OR tifluadom OR tilidine OR tonazocine) OR ABSTRACT(opioid* OR opiate* OR narcotic* OR alphaprodine OR nisentil OR prodine OR buprenorphine OR buprenorphine OR buprenex OR buprex OR prefin OR subutex OR temgesic OR "6029 m" OR 6029m OR rx6029m OR suboxone OR butorphanol OR dolorex OR moradol OR stadol OR torbugesic OR "bc 2627" OR bc2627 OR codeine OR ardinex OR idocodeine OR "n methylmorphine" OR hydrocodone OR codinovo OR dicodid OR dihydrocodeinone OR hycodan OR hycon OR hydrocodeinonebitartrate OR robidone OR oxycodone OR dihydrohydroxycodeinone OR dihydrone OR dinarkon OR eucodal OR oxiconum OR oxycodeinon OR oxycone OR oxycontin OR pancodine OR percocet OR theocodin OR vicodin OR dextromoramide OR "d moramide" OR palfium OR pyrrolamidol OR dextropropoxyphene OR propoxyphene OR "d propoxyphene" OR darvon OR enkephalin* OR dago OR dagol OR damge OR damgo OR "rx 783006" OR dpdpe OR fentanyl OR duragesic OR durogesic OR fentanest OR fentora OR phentanyl OR "r 4263" OR r4263 OR sublimaze OR alfenta OR alfentanil OR fanaxal OR limifen OR rapifen OR "r 39209" OR r39209 OR sufentanil* OR sulfentanil OR sulfentanyl OR "r 30730" OR r30730 OR hydromorphon* OR dihydromorphinone OR dilaudid OR laudacon OR palladone OR levorphanol OR levodroman OR levorphan OR "levo dromoran" OR "l dromoran" OR "medical heroin" OR meperidine OR demerol OR dolantin OR dolargan OR dolcontral OR dolin OR dolosal OR dolsin OR isonipecain OR lidol OR lydol OR operidine OR pethidine OR promedol OR dimethylmeperidine OR isopromedol OR trimeperidine OR meptazinol OR meptid OR "wy 22811" OR wy22811 OR methadone OR biodone OR dolophine OR metadol OR metasedin OR symoron OR methadose OR methex OR phenadone OR physeptone OR phymet OR pinadone OR amidone OR methaddict OR methadyl OR acetylmethadol OR alphacetylmethadol OR dimepheptanol OR levomethadyl OR levoacetylmethadol OR laam OR methadol OR orlaam OR acemethadone OR morphin* OR morphia OR duramorph OR "ms contin" OR morphia OR "oramorph sr" OR "sdz 202 250" OR sdz202250 OR "sdz202 250" OR nalbuphine OR nubain* OR "en 2234a" OR en2234a OR oxymorphone OR numorphan OR opana OR pentazocine OR fortral OR lexir OR talwin OR phenoperidine OR fenoperidine OR lealgin OR operidine OR "r 1406" OR r1406 OR pirinitramid* OR piritramid* OR dipidolor OR dipydolor OR tramadol OR adolonta OR amadol OR biodalgic OR biokanol OR contramal OR jutadol OR "k 315" OR k315 OR mtwtramadol OR nobligan OR prontofort OR "ranitidin 1a pharma5" OR takadol OR theradol OR tiral OR topalgic OR tradol OR tradolpuren OR tradonal OR tralgiol OR trama OR tramadorsch OR tramabeta OR tramadin OR tramadoc OR tramadoldolgit OR tramadolhameln OR tramadolor OR tramadolratiopharm OR tramadura OR tramagetic OR tramagit OR tramake OR tramal OR tramex OR tramundin OR trasedal OR ultram OR "xymel 50" OR zamudol OR zumalgic OR zydol OR zytram OR acetorophine OR acetylcodeine OR acetymethadol OR anileridine OR apadoline OR azidomorphine OR benzhydrocodone OR bezitramide OR bremazocine OR "brompton mixture" OR ciramadol OR cocomadol OR codydramol OR conorfone OR cyclazocine OR dextrorphan OR dezocine OR diamorphine OR diconal OR dihydroetorphine OR dihydromorphine OR dimethylthiambutene OR dipipanone OR dynorphin OR enadoline OR eptazocine OR ethylketazocine OR ethylmorphine OR etonitazene OR etorphine OR etoxeridine OR faxeladol OR furethidine OR gelonida OR isalmadol OR isomethodone OR ketazocine OR ketobemidone OR ketogan OR kyotorphin OR lefetamine OR levacetylmethadol OR levomethadone OR levorphanol OR metazocine OR methylsamidorphan OR tilidine OR nicodine OR nicomorphine OR noracymethadol OR "bufigen pentor nalbufin*" OR nalcryn OR nalpain OR onfor OR noracymethadol OR norbuprenorphine OR normorphine OR norpethidine OR norpropoxyphene OR nortramadol OR oliceridine OR oripavine OR pentamorphone OR phenadoxone OR phencyclidine OR picenadol OR piminodine OR piritramide OR profadol OR propiram OR sameridine OR samidorphan OR semorphone OR tapentadol OR thebaine OR tifluadom OR tilidine OR tonazocine) | 12511 |
| **3** | **#1 and #2 *[using search history tool]*** | **12** |

| **Google Search Used** | **Records Screened** | **Potentially Relevant Records** |
| --- | --- | --- |
| (electronic prescribing \| e-prescribing \| eprescribing) opioids filetype:pdf | Up to page 9 | 3 |
| (electronic prescribing \| e-prescribing \| eprescribing) opiates filetype:pdf | Up to page 6 | 0 |
| (electronic prescribing \| e-prescribing \| eprescribing) narcotics filetype:pdf | Up to page 7 | 0 |
| (electronic prescribing \| e-prescribing \| eprescribing) (buprenorphine \| suboxone) filetype:pdf | Up to page 3 | 1 |
| (electronic prescribing \| e-prescribing \| eprescribing) (codeine \| hydrocodone \| oxycodone \| oxycontin \| vicodin)) filetype:pdf | Up to page 2 | 0 |
| (electronic prescribing \| e-prescribing \| eprescribing) (dextropropoxyphene \| propoxyphene) filetype:pdf | Up to page 3 | 0 |
| (electronic prescribing \| e-prescribing \| eprescribing) (fentanyl \| alfentanil \| sufentanil) filetype:pdf | Up to page 5 | 0 |
| (electronic prescribing \| e-prescribing \| eprescribing) (hydromorphone \| dilaudid) filetype:pdf | Up to page 2 | 0 |
| (electronic prescribing \| e-prescribing \| eprescribing) (levorphanol) filetype:pdf | Up to page 3 | 0 |
| (electronic prescribing \| e-prescribing \| eprescribing) (meperidine \| promedol) filetype:pdf | Up to page 2 | 0 |
| (electronic prescribing \| e-prescribing \| eprescribing) (methadone \| methadyl) filetype:pdf | Up to page 3 | 0 |
| (electronic prescribing \| e-prescribing \| eprescribing) (morphine \| morphia) filetype:pdf | Up to page 3 | 0 |
| (electronic prescribing \| e-prescribing \| eprescribing) (oxymorphone \| pentazocine \| tramadol) filetype:pdf | Up to page 3 | 0 |
| **Included Resources: 1** | | |
